# Developing a new treatment for superficial fungal infection using antifungal Collagen‐HSAF dressing

**DOI:** 10.1002/btm2.10304

**Published:** 2022-03-10

**Authors:** Jing Zhong, Xiayi Yan, Xin Zuo, Xuan Zhao, Jiahui Yang, Qin Dou, Lulu Peng, Yuxiang Zhu, Yichen Xiao, Zeran Bian, Dalian He, Qiushuang Xu, Stephen Wright, Yaoyao Li, Liangcheng Du, Yan Wang, Jin Yuan

**Affiliations:** ^1^ State Key Laboratory of Ophthalmology, Zhongshan Ophthalmic Center Sun Yat‐sen University Guangzhou China; ^2^ College of Marine Life Sciences, and Institute of Evolution & Marine Biodiversity Ocean University of China Qingdao China; ^3^ Key Laboratory of Chemical Biology, School of Pharmaceutical Sciences Shandong University Jinan China; ^4^ Department of Chemistry University of Nebraska‐Lincoln Lincoln Nebraska USA

**Keywords:** *A. fumigatus* keratitis, antifungal dressing, cutaneous candidiasis, heat stable antifungal factor (HSAF), superficial fungal infections

## Abstract

Fungal pathogens are common causes of superficial clinical infection. Their increasing drug resistance gradually makes existing antifungal drugs ineffective. Heat stable antifungal factor (HSAF) is a novel antifungal natural product with a unique structure. However, the application of HSAF has been hampered by very low yield in the current microbial producers and from extremely poor solubility in water and common solvents. In this study, we developed an effective mode of treatment applying HSAF to superficial fungal infections. The marine‐derived *Lysobacter enzymogenes* YC36 contains the HSAF biosynthetic gene cluster, which we activated by the interspecific signaling molecule indole. An efficient extraction strategy was used to significantly improve the purity to 95.3%. Scanning electron microscopy images revealed that the Type I collagen‐based HSAF (Col‐HSAF) has a transparent appearance and good physical properties, and the in vitro sustained‐release effect of HSAF was maintained for more than 2 weeks. The effective therapeutic concentration of Col‐HSAF against superficial fungal infection was explored, and Col‐HSAF showed good biocompatibility, lower clinical scores, mild histological changes, and antifungal capabilities in animals with *Aspergillus fumigatus* keratitis and cutaneous candidiasis. In conclusion, Col‐HSAF is an antifungal reagent with significant clinical value in the treatment of superficial fungal infections.

**Abbreviations**
ATCC: American Type Culture Collection
CFU: colony‐forming unit
CFW: calcofluor white
CGMCC: China General Microbiological Culture Collection Center
Col‐HSAF: Type I collagen‐based HSAF
CT: cycle threshold
FK: *A. fumigatus* keratitis
GDMCC: Guangdong Microbiological Culture Collection Center
HCECs: human corneal epithelial cells
HE: hematoxylin‐eosin
HPLC: high‐performance liquid chromatography
HRMS: high‐resolution mass spectrometry
HSAF: heat stable antifungal factor
MIC: minimum inhibitory concentration
PBS: phosphate‐buffered saline
PDA: potato dextrose agar
SDA: Sabouraud dextrose agar
TPM: two‐photon microscopy

## 1 INTRODUCTION

Fungi infect billions of people every year, yet the contribution of fungi to the global burden of disease is largely unrecognized. Although true mortality rates are unknown because of a lack of good epidemiological data, the incidence of invasive fungal infections is rising as a result of modern medical interventions and immunosuppressive diseases.^1^ Of the estimated 5 million or more fungal species worldwide, approximately 300 fungal species have been documented as causing human disease, but only 20–25 do so on a relatively frequent basis, including the fungal species *A. fumigatus* and *Candida krusei*.^2^ Compared with antibacterial research, the development of new antifungal drugs has been relatively slow, and there are only four major classes of antifungal agents (polyenes, flucytosine, azoles, and echinocandins).^3^ For example, among the clinically common antifungal agents, amphotericin B (AMB) and natamycin (E235) belong to the polyene class which can destroy fungal cell walls, while voriconazole is the azole class that can exert an antifungal effect by inhibiting ergosterol production. A number of hurdles to antifungal drug development must be overcome, one of which is the problem of co‐toxicity for eukaryotic targets: as fungi are eukaryotic pathogens with a number of important similarities with their human hosts, antifungals often exhibit unacceptably high toxicity to human cells, which complicates the search for antifungal targets and slows the development of antifungal agents.^4^ Over the past 5–6 years, with the widespread use of antifungal agents, there has been a clear increase in the identification of yeasts that have developed resistance.^3^ Therefore, promising new antifungal agents are urgently needed to meet clinical needs.

HSAF isolated from *L. enzymogenes* strains has exhibited broad‐spectrum antifungal activity.^5^, ^6^, ^7^ Previously, HSAF was used for the biological control of plant fungal diseases by interfering in the biosynthesis of fungal sphingolipids, a mechanism that differs from that of other antifungal drugs.^8^ Its chemical structure contains a tetramic acid moiety and a 5,5,6‐tricyclic skeleton, which is different from any existing antifungal drug. Due to this complex structure, it is extremely difficult to obtain HSAF through organic chemical synthesis. At present, HSAF can be produced only by *Lysobacter*, and its biosynthetic gene cluster and synthesis mechanism have been gradually revealed.^6^, ^7^ The biosynthetic genes of HSAF include a single module of PKS/NRPS, arginase, 5 redox enzymes, and sterol desaturation/fatty acid hydroxylase.^9^, ^10^, ^11^ To date, HSAF has not been synthesized by chemical means, and *L. enzymogenes* is one of the few strains that can produce HSAF. The regulatory mechanism of HSAF biosynthesis and how to improve the yield of HSAF are still largely unknown. HSAF has been used in only agricultural fungal diseases since its discovery. As HSAF has a novel chemical structure and antifungal mechanism, solving the shortcomings of low yield, inadequate purity, and low solubility would greatly increase HSAF's therapeutic value and broaden its application scope.

In combination with its relatively poor solubility even in organic solvents, the cytotoxicity of HSAF in organic solvents has prevented its extension into the clinic.^12^ The discovery and development of new drugs are not sufficient to achieve therapeutic excellence and enter the drug market. The topical delivery of antifungal drugs is perhaps the best treatment for superficial fungal infections; its advantages include site‐specific drug delivery, decreased systemic toxicity, increased efficacy of treatment, and improved bioavailability.^13^ Conventional formulations require high dosages and repeated administration, associated with an increased risk of both local and systemic toxicity. A new drug delivery system for insoluble HSAF is necessary to reduce local side effects and increase therapeutic efficacy.^14^ Hydrogels are promising materials for drug delivery and wound healing applications due to their good biodegradability, low toxicity, and well‐known safety profile.^15^ On skin contact, these formulations form a semi‐occlusive film over the skin and release the drug in a controlled manner, which is ideal for the topical delivery of HSAF.^16^

In this study, we identified a novel strategy for the treatment of superficial fungal infections with HSAF. We optimized an HSAF purification technology by using marine‐derived *L. enzymogenes* YC36, in which HSAF production was induced by indole, a ubiquitous interspecific signaling molecule. Then, we constructed collagen‐based HSAF composite membranes by choosing Type I collagen (Col‐HSAF) as the carrier, established animal models of two superficial fungal infections (*A. fumigatus* keratitis and cutaneous candidiasis), and documented the excellent biocompatibility, antifungal activity and clinical therapeutic effects of Col‐HSAF composite membranes.

## 2 RESULTS

### 2.1 Extraction and antifungal activity of HSAF

**FIGURE 1:**
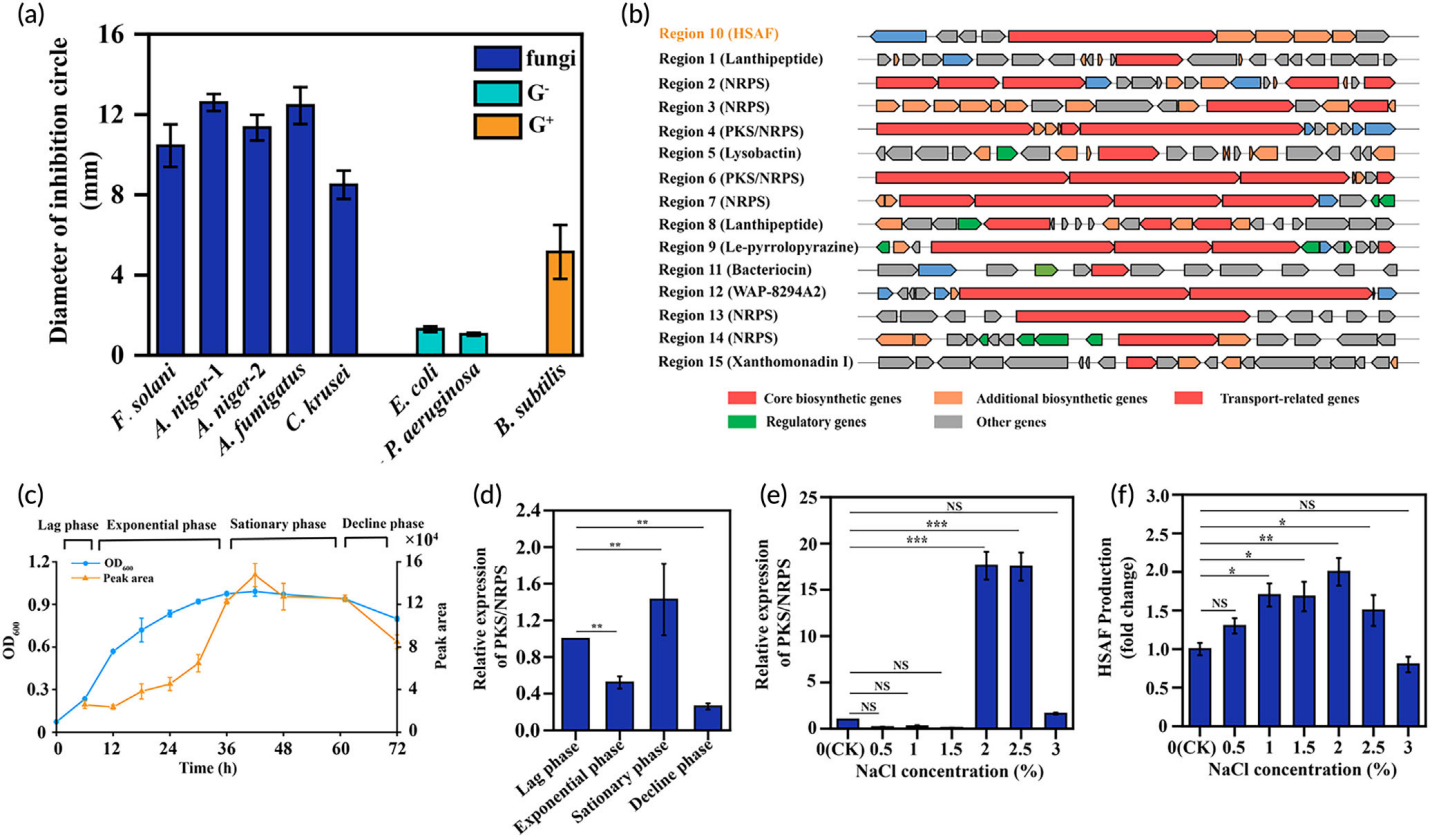
*L. enzymogenes* YC36 inhibits the growth of bacteria and fungi. (a) Effects of *L. enzymogenes* YC36 on the growth of selected fungi and gram‐negative and gram‐positive bacteria. Fungi include *F. solani* ATCC 36031, *A. niger* ATCC 16404, *A. niger* CMCC 98003 (f), *A. fumigatus* AS 3.1320, and *C. krusei* ATCC 14243. Gram‐negative bacteria include *E. coli* ATCC 25923 and *P. aeruginosa* PAO1, and gram‐positive bacteria include *B. subtilis* 168. (b) Analysis of 15 secondary metabolite gene clusters in *L. enzymogenes* YC36. (c) The growth and peak area of HSAF in *L. enzymogenes* YC36 at 0–72 h. The lag phase: 0–6 h, the exponential phase: 6–36 h, the stationary phase: 36–60 h, and the decline phase: 60–72 h. (d) The relative expression levels of PKS/NRPS at different phases in *L. enzymogenes* YC36. The lag phase was selected as the control experiment. (e) The relative expression levels of PKS/NRPS in *L. enzymogenes* YC36 cultured with different concentrations of NaCl. The 0% NaCl concentration was selected as the control for the experiment. (f) The effect of different NaCl concentrations on HSAF production in *L. enzymogenes* YC36. The 0% NaCl concentration was selected as the control for the experiment. NS, not significant, *p < 0.05, **p < 0.01, and ***p < 0.001, replicates = 3. All data are presented as means ± SEM, and statistical significance was determined using paired *t* tests

**FIGURE 2:**
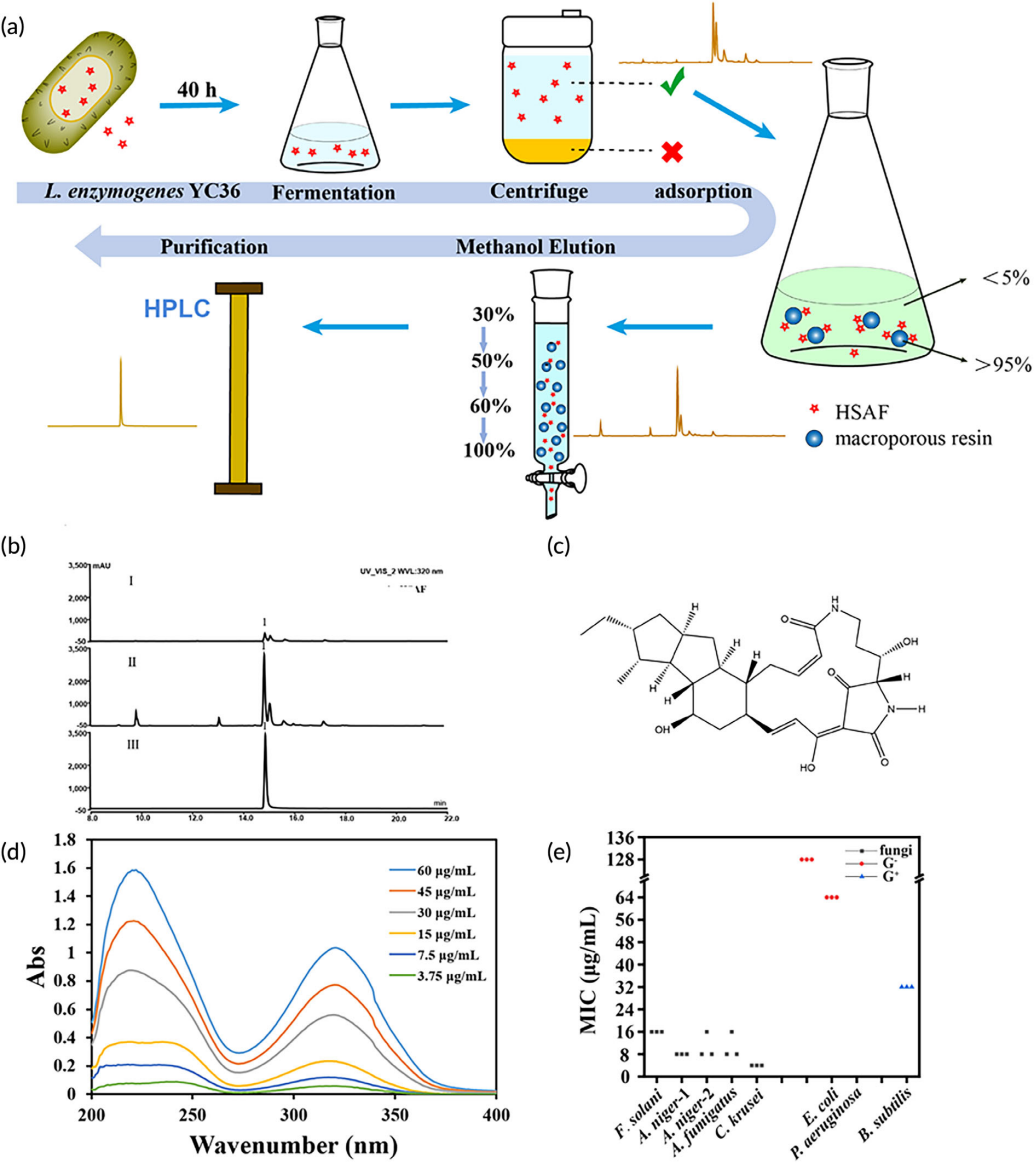
HSAF extraction from *L. enzymogenes* YC36 and activity. (a) Schematic diagram of the HSAF purification process. (b) HPLC detected three samples with different purification degrees during HSAF extraction. Supernatant of bacterial fluid (I), 100% methanol eluent (II), purified (III). (c) The structure of HSAF. (d) (dA) UV absorption curve of HSAF from 200 to 400 nm. (e) The MIC of HSAF against fungi. Three strains of bacteria were selected as the control conditions. The results show that the antifungal effect of HSAF was much stronger than the antibacterial effect. NS, not significant, *p <0.05, **p <0.01, and ***p <0.001, replicates =3. All data are presented as means ± SEM, and statistical significance was determined using paired *t* tests

*L. enzymogenes* YC36 strain was isolated offshore of Qingdao. The results showed that *L. enzymogenes* YC36 has a remarkable inhibitory effect on fungi, such as *Fusarium solani* ATCC 36031, *Aspergillus niger* ATCC 16404, *A. niger* CMCC (F) 98003. *Aspergillus fumigatus* AS 3.1320, and *C. krusei* ATCC 14243. It also has a certain inhibitory effect on gram‐negative bacteria, such as *Escherichia coli* ATCC 25923 and *Pseudomonas aeruginosa* PAO1. Its inhibitory level against gram‐positive bacteria, such as *Bacillus subtilis* 168, is weak (Figure 1a). We explored the biological mechanism by analyzing the genome of *L. enzymogenes* YC36, which revealed 15 secondary metabolite biosynthetic gene clusters, including the antifungal compound HSAF (Figure 1b). We investigated the production of HSAF in *L. enzymogenes* YC36 with bacterial growth. HSAF starts to be produced in the lag phase, but production is very low in the first 36 h and peaks from 36–48 h, followed by a gradual decrease in production (Figure 1c). In the stationary phase, the expression of PKS/NRPS in *L. enzymogenes* YC36 increased significantly (Figure 1d). In addition, 2% NaCl promoted the expression of PKS/NRPS and the production of HSAF (Figure 1e,f), probably because 2% NaCl is most closely to the original growth environment of *L. enzymogenes* YC36. The efficient and high‐purity extraction of HSAF is one of the bottlenecks restricting its subsequent research. The HSAF purification process is shown in Figure 2a as a schematic diagram. Initially, the purity of HSAF in the supernatant of bacterial liquid was 59.28% according to high‐performance liquid chromatography (HPLC) data. The microporous resin was used to adsorb HSAF in the supernatant. The adsorption rate was >95%, and residual HSAF was rarely detected in the culture medium (Figure S1). Subsequently, after eluting the impurities from the microporous resin with low to high methanol concentrations, the purity of HSAF collected in 100% methanol increased to 68.46%, and the recovery was 70.55%. HPLC is the final step in HSAF purification, the recovery rate of HPLC was 52.48% (Figure 2b), and the ultimate purity of HSAF increased to 95.3% (Figures S2 and S3). Figure 2c,d shows the structure and UV absorption spectrum of HSAF. HSAF was produced at a concentration of approximately 7 mg/L. The minimum inhibitory concentration (MIC) of HSAF against fungi was 4–16 μg/ml (Figure 2e).

### 2.2 Stimulation of HSAF production by indole

**FIGURE 3:**
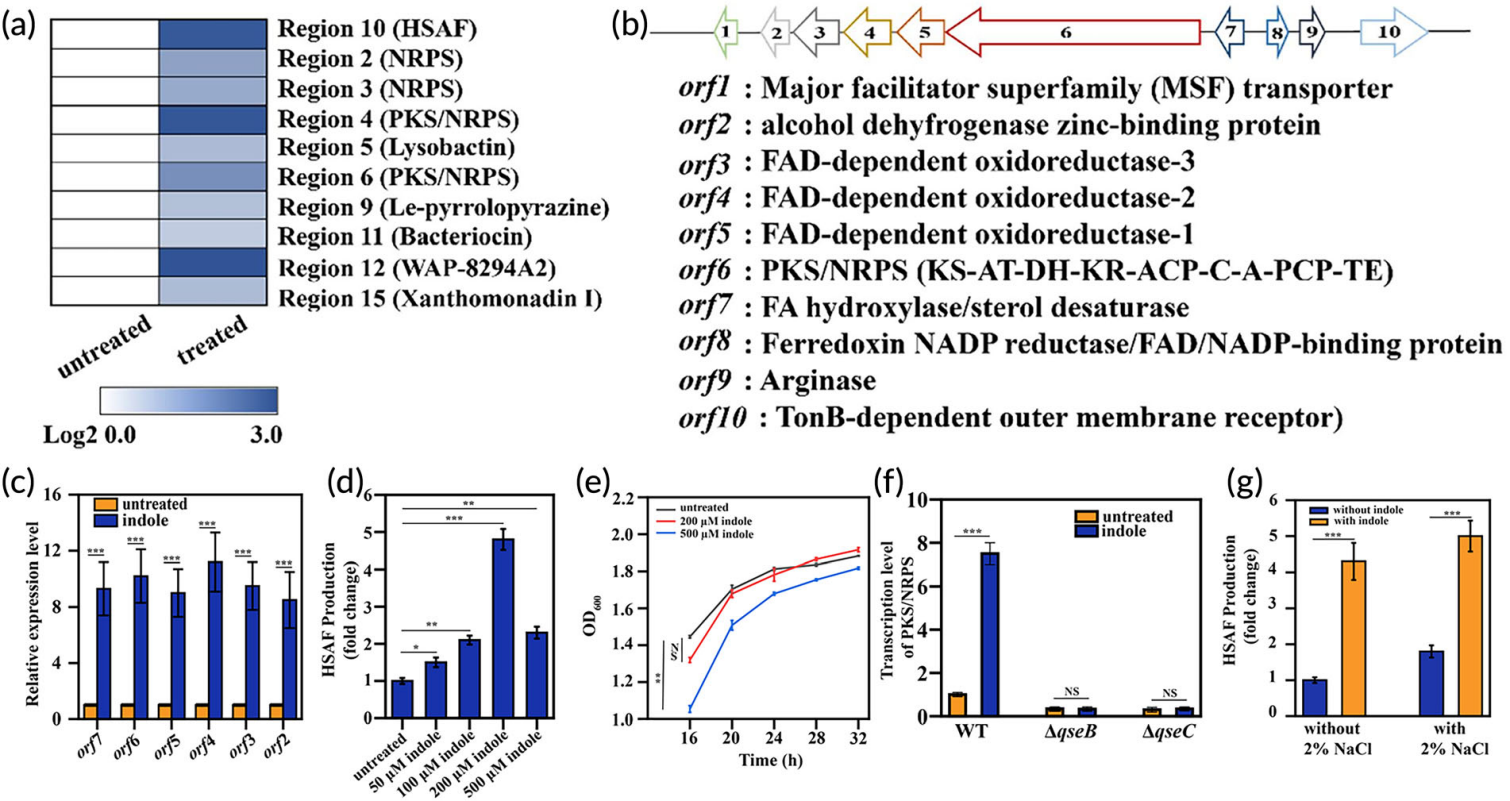
The production of HSAF can be induced by adding indole. (a) Heatmap showing the relative transcription levels of the secondary metabolite biosynthesis gene clusters with indole. (b) The HSAF gene cluster of *L. enzymogenes* YC36, which includes 10 genes. (c) Real‐time PCR assays of the relative expression levels of the six core genes *orf*2‐7 involved in HSAF biosynthesis. (d) The effect of exogenous indole on HSAF production in *L. enzymogenes*. Indole concentrations of 50, 100, 200, 500 μM. The HPLC peak area was calculated to quantify the production of HSAF. (e) Growth curves of *L. enzymogenes* YC36 in the presence of 200 or 500 μM indole. The control group was not cultured with indole. (f) Effect of the deletion of the QseC/QseB system on HSAF gene expression in *L. enzymogenes* YC36. The expression level of the PKS/NRPS gene was analyzed using real‐time PCR with indole. (g) The effect of the combination of 2% NaCl concentration and 200 μM indole on HSAF production. NS, not significant, *p <0.05, **p <0.01, and ***p <0.001, replicates = 3. All data are presented as means ± SEM, and statistical significance was determined using paired *t* tests

As mentioned above, the low yield of HSAF is one of the bottlenecks affecting its subsequent application. In this study, we were surprised to find that indole, a signaling molecule, significantly improved the expression of approximately two‐thirds of the biosynthesis gene clusters of secondary metabolites in *L. enzymogenes* YC36 (Figure 3a). It is worth noting that the expression of the HSAF gene cluster was upregulated by 9‐fold, while the expression of the WAP‐8294A2 gene cluster was upregulated by 10‐fold. The HSAF biosynthesis gene cluster contains 10 *orfs*. They include major facilitator superfamily transporters (*orf1*), alcohol dehydrogenase zinc‐binding protein (*orf2*), FAD‐dependent oxidoreductase‐3 (*orf3*), FAD‐dependent oxidoreductase‐2 (*orf4*), FAD‐dependent oxidoreductase‐1 (*orf5*), PKS/NRPS (*orf6*), FA hydroxylase/sterol desaturase (*orf7*), ferredoxin NADP reductase/FAD/NADP‐binding protein (*orf8*), arginase (*orf9*), and TonB‐dependent outer membrane receptor (*orf10*) (Figure 3b). Real‐time PCR results showed that the addition of exogenous indole markedly upregulated the 6 core genes in the HSAF gene cluster, and *orf2‐7* expression was upregulated 8‐11‐fold (Figure 3c). Indole regulated HSAF production in a dose‐dependent manner. Indole (50 μM) increased HSAF production by approximately 50% compared with untreated *L. enzymogenes* YC36, 100 μM indole increased the production by approximately one‐fold, 200 μM indole increased it by approximately 4‐fold, and 500 μM indole increased production by approximately 1.5‐fold (Figure 3d). Therefore, 200 μM indole was selected for subsequent experiments to increase HSAF production and did not affect the growth speed of *L. enzymogenes* YC36 (Figure 3e). The two‐component system QseC/QseB was shown to be sensitive to indoles.^17^ If *qseC/qseB* was knocked out, PKS/NRPS expression was significantly downregulated, regardless of the presence or absence of indole (Figure 3f). In addition, the combination of 2% NaCl and 200 μM indole exerted a greater effect on improving the HSAF yield than the addition of a single molecule, which is important for future studies on increasing HSAF production (Figure 3g).

### 2.3 Synthesis of Col‐HSAF and property characterization

**FIGURE 4:**
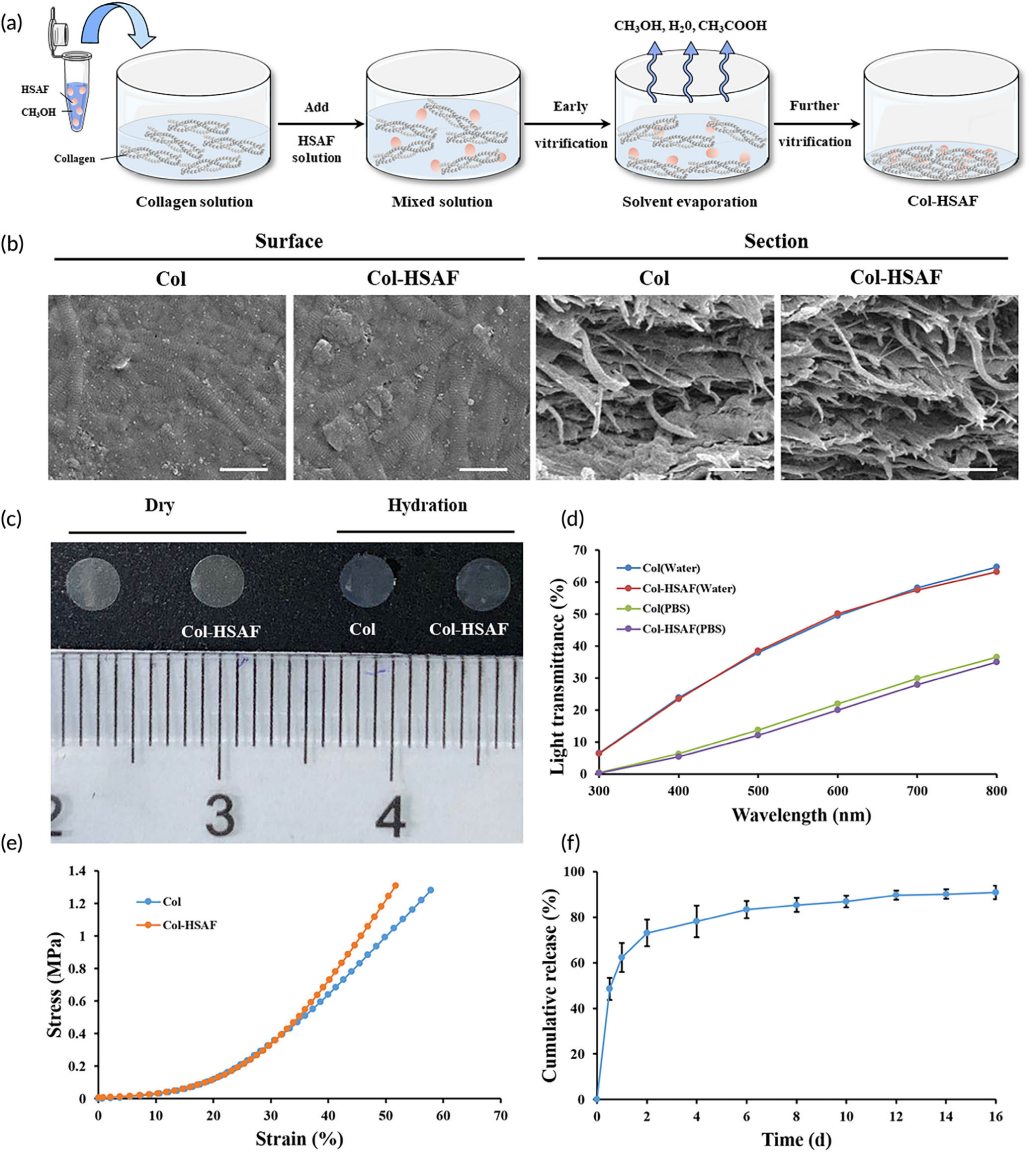
Preparation and characterization of collagen‐based membranes loaded with HSAF. (a) Schematic of Col‐HSAF preparation. (b) Scanning electron micrographs of Col and Col‐HSAF surfaces and sections (scale bar =1 μm). (c) Gross comparison of Col and Col‐HSAF before and after hydration. (d) Transmittance of Col and Col‐HSAF after rehydration in deionized water and PBS solution. (e) Stress–strain curve of Col and Col‐HSAF. (f) Cumulative release of HSAF from Col‐HSAF. NS, not significant, *p < 0.05, **p < 0.01, and ***p < 0.001, replicates = 3. All data are presented as means ± SEM, and statistical significance was determined using paired *t* tests

HSAF is insoluble in water and is difficult to use clinically as a topical antifungal agent for its biotoxicity, as shown in our preliminary experiment (Figure S4). To solve this problem, we chose Type I collagen as the carrier and constructed collagen‐based HSAF composite membranes (Figure 4a). Scanning electron microscopy(SEM) images revealed that the prepared Col‐HSAF retains the unique fibrous structure of collagen (Figure 4b), and Col‐HSAF has a similar appearance and physical properties to the unmodified collagen membrane. Col‐HSAF is a thin translucent membrane and can be cut into a predetermined shape, such as a circle, according to the requirements, and the dried sample can be quickly rehydrated by soaking in water before use without losing its shape (Figure 4c). Compared with rehydration in phosphate‐buffered saline (PBS) solution, Col‐HSAF rehydration in deionized water resulted in better optical transparency, and the light transmittance at 800 nm wavelength reached 70% (Figure 4d). Regarding the mechanical properties, the tensile strength of Col‐HSAF was 1.30 MPa, similar to that of Col (1.28 MPa) (Figure 4e). Col‐HSAF also showed an appropriate elongation at break (51.71%), and was sufficiently strong to withstand surgical operation and the ocular surface friction in subsequent in vivo experiments. To characterize the sustained‐release curve of HSAF in Col‐HSAF, we first scanned the absorbance of HSAF at 200–400 nm with an ultraviolet spectrophotometer and determined the appropriate absorption peak at 320 nm. A standard curve was established (Figure S5), and an in vitro sustained‐release curve of HSAF was finally determined and drawn (Figure 4f). The in vitro sustained‐release effect of HSAF was maintained for more than 2 weeks.

### 2.4 Cytotoxicity, cell apoptosis, and antifungal effects of Col‐HSAF

**FIGURE 5:**
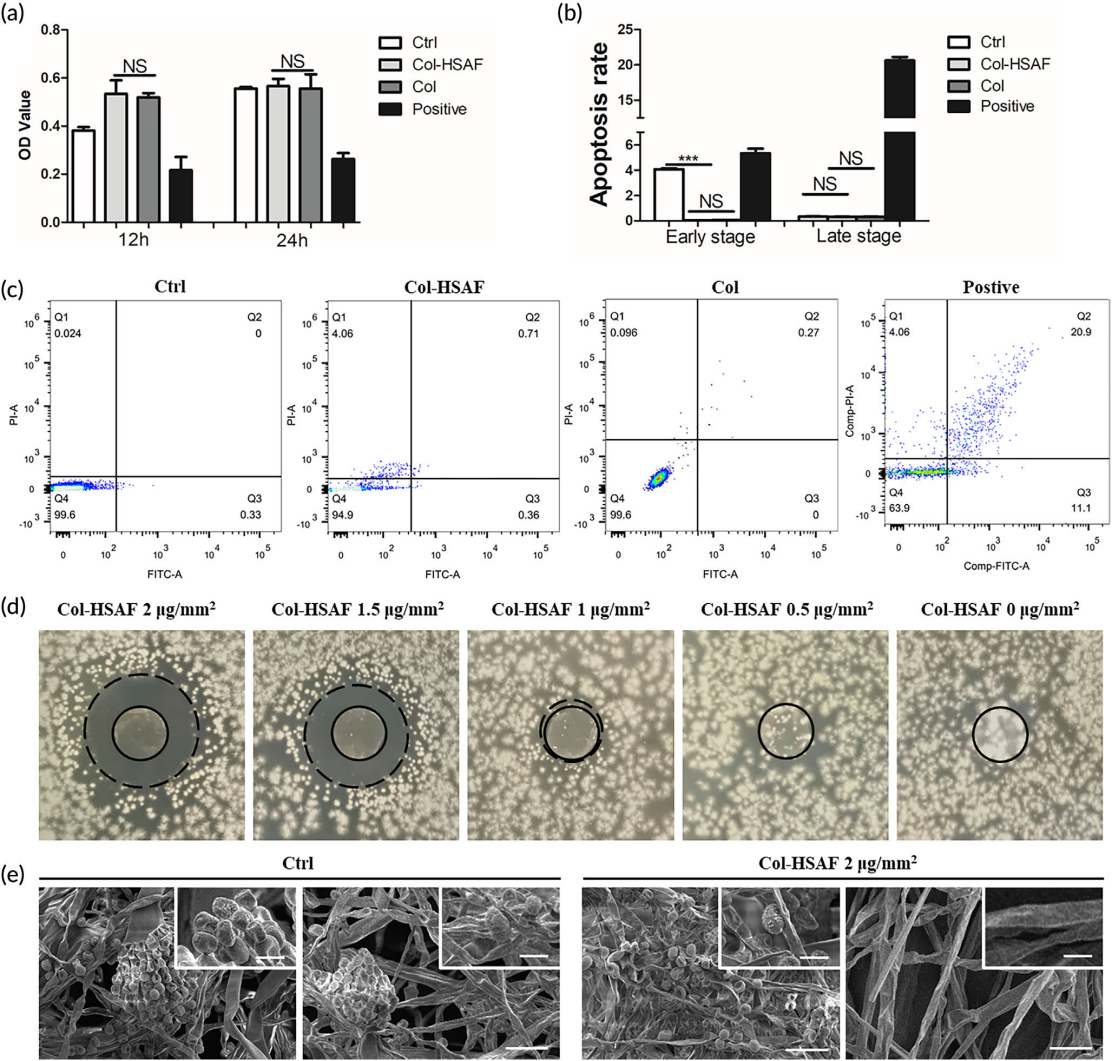
The proliferation of HCECs. (a) In a Cell Counting Kit‐8 assay, HCECs were treated with PBS, Col‐HSAF and 0.2 M NaCl for 12 and 24 h to assay their cell viability. (b) In the FITC‐Annexin‐V/PI labeling experiment, HCECs were treated with Col‐HSAF and PBS for 24 h. (c) The inhibition zone of Col‐HSAF against *A. fumigatus* at 2, 1.5, 1 and 0 μg/mm^2^. (d) Scanning electron micrographs of *A. fumigatus* treated without/with HSAF at different positions. (Scale bar in larger photos is 50 μm, scale bar in smaller photos is 10 μm). NS, not significant, *p < 0.05, **p < 0.01, and ***p < 0.001, *n* =5 samples per group. All data are presented as means ± SEM, and statistical significance was determined using paired *t* tests

Human corneal epithelial cells (HCECs) were co‐cultured with the collagen membrane, Col‐HSAF, and 0.2 M NaCl (positive control) for 24 h to evaluate and compare the cytotoxic effects of HSAF on inducing apoptosis in HCECs. Compared with the control (Ctrl) group, the coculture group showed similar cytocompatibility, with optical density (OD) values of 0.344 ± 0.007,0.534 ± 0.062, 0.520 ± 0.017, and 0.217 ± 0.055, respectively, for the Ctrl, Col‐HSAF, Col, and positive groups at 12 h, respectively; and OD values of 0.548 ± 0.007, 0.560 ± 0.061, 0.557 ± 0.059, and 0.263 ± 0.024, respectively, at 24 h (Figure 5a). Flow cytometry (Figure 5b,c) revealed that the cell apoptosis rate (0.024 ± 0.01% early apoptotic cells and 0.33 ± 0.02% late apoptotic cells) of the Col‐HSAF group was not as high as that of the Ctrl group (4.06 ± 0.03% early apoptotic cells and 0.38 ± 0.04% late apoptotic cells), and lower obviously than the positive group (5.34 ± 0.37% early apoptotic cells and 20.62 ± 0.50% late apoptotic cells).

The results of the antifungal zone of inhibition showed that the size of the inhibition zone of Col‐HSAF increased with the increase in the HSAF capacity, which was 11.40 ± 0.47, 10.18 ± 0.16, 6.25 ± 0.21, and 0 mm for the 2, 1.5, 1, and 0 μg/mm^2^ Col‐ HSAF groups. When the HSAF content was <1 μg/mm^2^, it had no obvious antifungal effect (Figure 5d). Four antibiotics natamycin (E235), amphotericin B (AMB), voriconazole (VCA), and clotrimazole (CBD) commonly used in the treatment of fungal keratitis were selected to compare antifungal efficacy with HSAF. MIC ranges and mean values of HSAF, voriconazole, natamycin, amphotericin B, and clotrimazole were 7.8–15.6 and 2.0–4.0, 1.0–2.0 and 0.5–1.0, 0.1–0.3 and 0.06–0.1, >16 and 8.0–16.0, and <0.03 and 4.0–8.0 μg/ml for *A. fumigatus* and *C. krusei* (Table 1; Figure S6). A transmission electron micrograph showed that the density of fungal hyphae was obviously richer and the length was longer in the Ctrl group than in the Col‐HSAF group, and fewer spores were observed in the Col‐HSAF group (Figure 5e).

**TABLE 1:** MIC assay for different antifungal drugs

	*A. fumigatus* (μg/ml)	*C. krusel* (μg/ml)
HSAF	7.8–15.6	2.0–4.0
Natamycin (E235)	1.0–2.0	0.5–1.0
Voriconazole (VCA)	0.1–0.3	0.06–0.1
Amphotericin B (AMB)	>16	8.0–16.0
Clotrimazole (CBD)	<0.03	4.0–8.0

### 2.5 Therapeutic effects of Col‐HSAF on *A. fumigatus* keratitis and cutaneous candidiasis

Mouse models of two fungal infections were constructed to further evaluate the inhibitory effects of Col‐HSAF on fungal proliferation in vivo: *A. fumigatus* keratitis (FK) and cutaneous candidiasis.

The pharmacokinetics of topical Col‐HSAF in rabbits are shown in Table 2, the apparent elimination half‐life (*t*_1/2_) values were 213.150 ± 12.281, 541.995 ± 782.876, and 0 h, and the time‐to‐maximum concentration (*T*_max_) values were 3.75 ± 1.50, 3.00 ± 0.00, and 3.00 ± 0.00 h in the conjunctiva, cornea and aqueous humor, respectively. The highest maximum concentration (*C*_max_) was detected in the conjunctiva at 1055.894 ± 648.338 ng/ml, followed by 493.759 ± 524.015 and 99.427 ± 132.680 ng/ml in the cornea and aqueous humor, respectively. The area under the curve (AUC) (0–*t*) and mean residence time (MRT) (0–*t*) of Col‐HSAF were 18220.101 ± 6937.698, 4172.306 ± 1662.436, and 370.671 ± 434.858 ng·h·ml^−1^ and 86.421 ± 20.697, 60.317 ± 69.685, and 4.849 ± 1.648 h in the conjunctiva, cornea and aqueous humor, respectively (Table 2). After the topical application of Col‐HSAF, HSAF was distributed within various tissues at different times. The conjunctiva showed the highest value among tissues from all the subgroups at 3, 6, 24, 72, 168, and 336 h after topical administration (Figure S7).

**TABLE 2:** Primary pharmacokinetic parameters of Col‐HSAF in rabbit eyes

Tissue	*t*_1/2_ (h)	*C*_max_ (ng/ml)	AUC (0‐*t*) (ng·h·ml^−1^)	MRT (0‐*t*) (h)
Conjunctiva	213.150 ± 12.281	1055.894 ± 648.338	18220.101 ± 6937.698	86.421 ± 20.697
Cornea	541.995 ± 782.876	493.759 ± 524.015	4172.306 ± 1662.436	60.317 ± 69.685
Aqueous humor	–	99.427 ± 132.680	370.671 ± 434.858	4.849 ± 1.648

**FIGURE 6:**
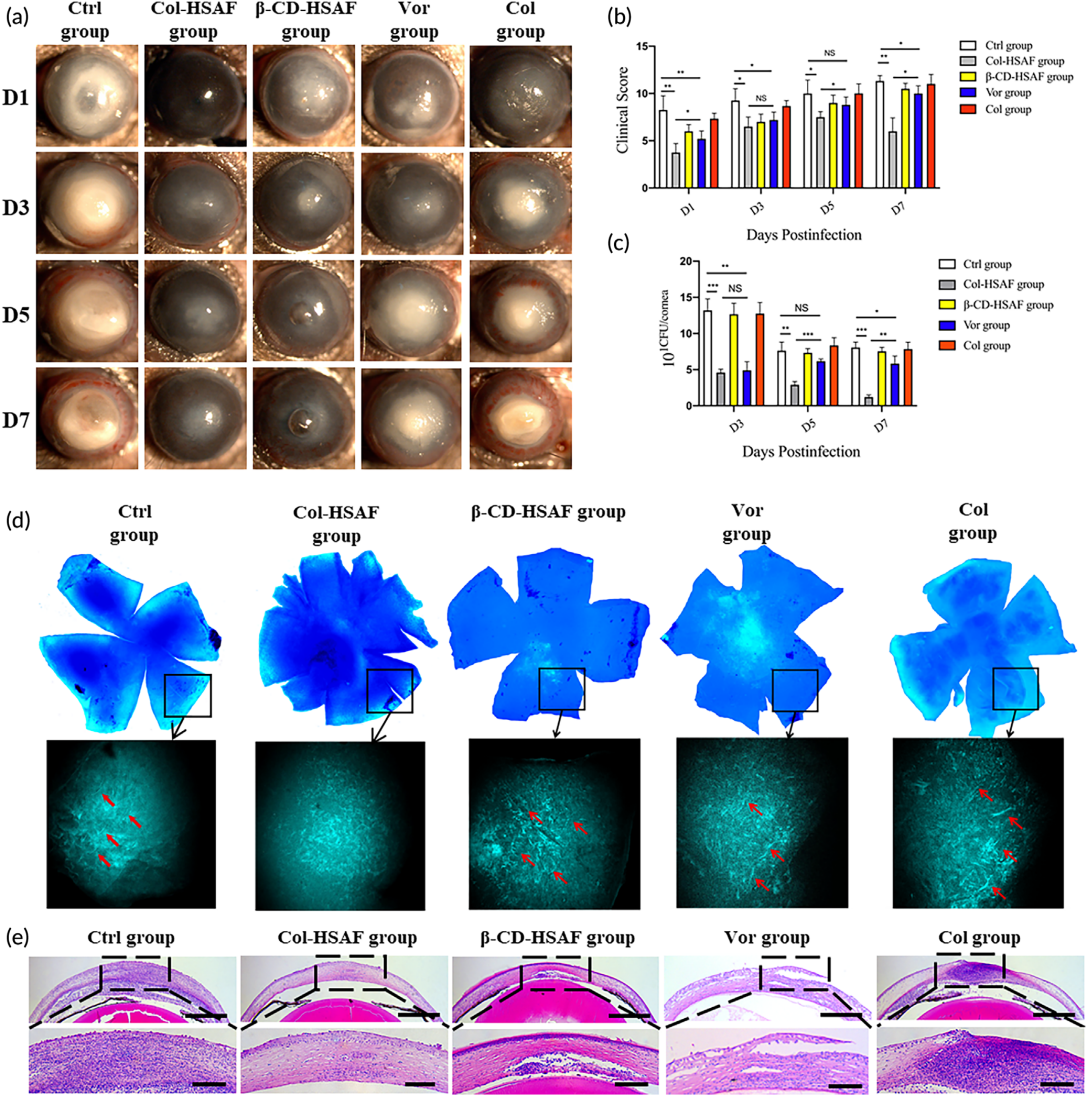
(a) Representative ocular images and (b) clinical scores are shown to indicate disease progression in the Ctrl, Col‐HSAF, β‐CD‐HSAF, Vor, and Col groups in the FK animal model at 1, 3, 5 and 7 days. (c) Viable fungal loads reported in log colony‐forming units (CFUs) in the Ctrl, Col‐HSAF, β‐CD‐HSAF, Vor, Col groups. (d) Image of petaloid fluorescence staining after staining with KOH and CFW (left image, magnification 40×) and TPM image (right image, magnification 800×) showing the fungal distribution in the mouse corneas treated with Ctrl, Col‐HSAF, β‐CD‐HSAF, Vor, and Col group. Red arrowheads: filamentous hyphae. (e) The histopathology of the Ctrl, Col‐HSAF, β‐CD‐HSAF, Vor, Col groups at 5 days. Magnification: 16× (slit‐lamp photographs) and 40× (HE staining). NS, not significant, *p < 0.05, **p < 0.01, and ***p < 0.001, *n* =5 animals per group. All data are presented as means ± SEM, and statistical significance was determined using paired *t* tests

For the *A. fumigatus* keratitis model, the Col/Col‐HSAF membranes and voriconazole (Vor)/β‐CD‐HSAF solution were used to treat the eyes of mice with FK. The therapeutic effects were observed under a clinical slit lamp for seven consecutive days, and representative images of mouse eyes at 1, 3, 5, and 7 days are depicted (Figures 6a and S8a,b). The ocular images of the healthy animals (BK group) showed that the full‐thickness cornea was transparent and intact, and the iris was clearly visible with no new blood vessels present in the limbus. As shown in Figure 6b, compared with the Ctrl, β‐CD‐HSAF, Col groups, the other two groups treated with the drug showed a significant therapeutic effect on keratomycosis induced by *A. fumigatus* infection. On days 1, 5, and 7, eyes treated with Col‐HSAF (3.8 ± 0.8, 6.5 ± 0.9, 7.5 ± 0.5, and 6 ± 1.2) showed lower clinical scores than the Col, Ctrl, and Vor groups which is as the positive control (*p* <0.05), and a nonsignificant difference was observed between the Col‐HSAF and Col‐Vor groups (3.6 ± 0.8, 6.5 ± 1.1, 7 ± 1.6, and 5.8 ± 1.5) (Figure S8c). In particular, an increasing trend of the clinical scores was observed in the Ctrl (8.3 ± 1.3, 9 ± 1.4, 10 ± 1.2, and 11.3 ± 0.5), β‐CD‐HSAF (6 ± 0.7, 7 ± 0.8, 9 ± 0.8, and 10.5 ± 0.6), Vor (5.2 ± 0.7, 7.2 ± 0.7, 8.8 ± 0.7, and 9.3 ± 0.9), and Col groups (7.3 ± 0.5, 8.7 ± 0.5, 10 ± 0.8, and 11 ± 0.8) over time, while a decreasing trend appeared in the Col‐HSAF groups from day 5 to day 7.

In addition, a fungal loading test (Figure 6c) was performed to detect the quantity and vitality of live fungi in the cornea and to explore the effect of Col‐HSAF on inhibiting fungal proliferation in vivo. Compared with the viable fungal infiltration of the Ctrl groups (13.2 ± 1.3, 7.6 ± 1.2, 8.1 ± 0.6), the viable fungal infiltration of the Col‐HSAF‐treated group (6 ± 0.4, 2.9 ± 0.4, 1.2 ± 0.2) and Col‐Vor‐treated group (4.6 ± 0.4, 2.9 ± 0.6, 1.0 ± 0.2) (Figure S8d) was significantly reduced on days 3, 5, and 7 (*p* <0.01). The viable fungal infiltration of the Vor‐treated group (4.9 ± 1.0, 6.2 ± 0.2, 5.8 ± 0.9) was higher than the Col‐HSAF‐treated group on days 5 and 7 (*p* <0.01). Additionally, the β‐CD‐HSAF group (12.7 ± 1.2, 7.3 ± 0.5, and 7.5 ± 0.4) and Col group (12.7 ± 1.3, 8.3 ± 0.9, and 7.8 ± 0.8) were not significantly different from the Ctrl group over time. The fungal hyphae in the cornea were tested ex vivo by performing fluorescence staining after staining with KOH and calcofluor white (CFW), and two‐photon microscopy (TPM) was used to image the corneas in the FK mouse models (Figures 6d and S8e) on the 3rd day postinfection. A cluster of fungal hyphae with short rod shapes was observed in the perimeter zone of the cornea in the Ctrl, β‐CD‐HSAF, and Col groups, and fewer fungal hyphae were observed in the perimeter zone of the cornea in the Vor group. No obvious fungal hyphae were observed in the Col‐HSAF and Col‐Vor groups. In the TPM images, obvious filamentous hyphae in the stromal layers (marked with red arrowheads) were observed in the Ctrl group, and hyphal structures were distinctly identified 50 μm from the surface. Images of the stroma are shown for each group, which showed a much denser cell distribution in the cornea of the Ctrl group than in the Col‐HSAF group, indicating severe infection. Most cells were irregularly distributed and were difficult to identify on the basis of cell morphology and distribution because of high cell density. The histological evaluation of Col‐HSAF was achieved by performing hematoxylin and eosin (H&E) staining of corneal slices. Corneal stromal edema and infiltration of inflammatory cells in Col‐HSAF‐treated and Col‐Vor‐treated corneal specimens were significantly decreased on days 5 after infection compared with those in the Ctrl, β‐CD‐HSAF, Vor, and Col groups (Figures 6e and S8f).

**FIGURE 7:**
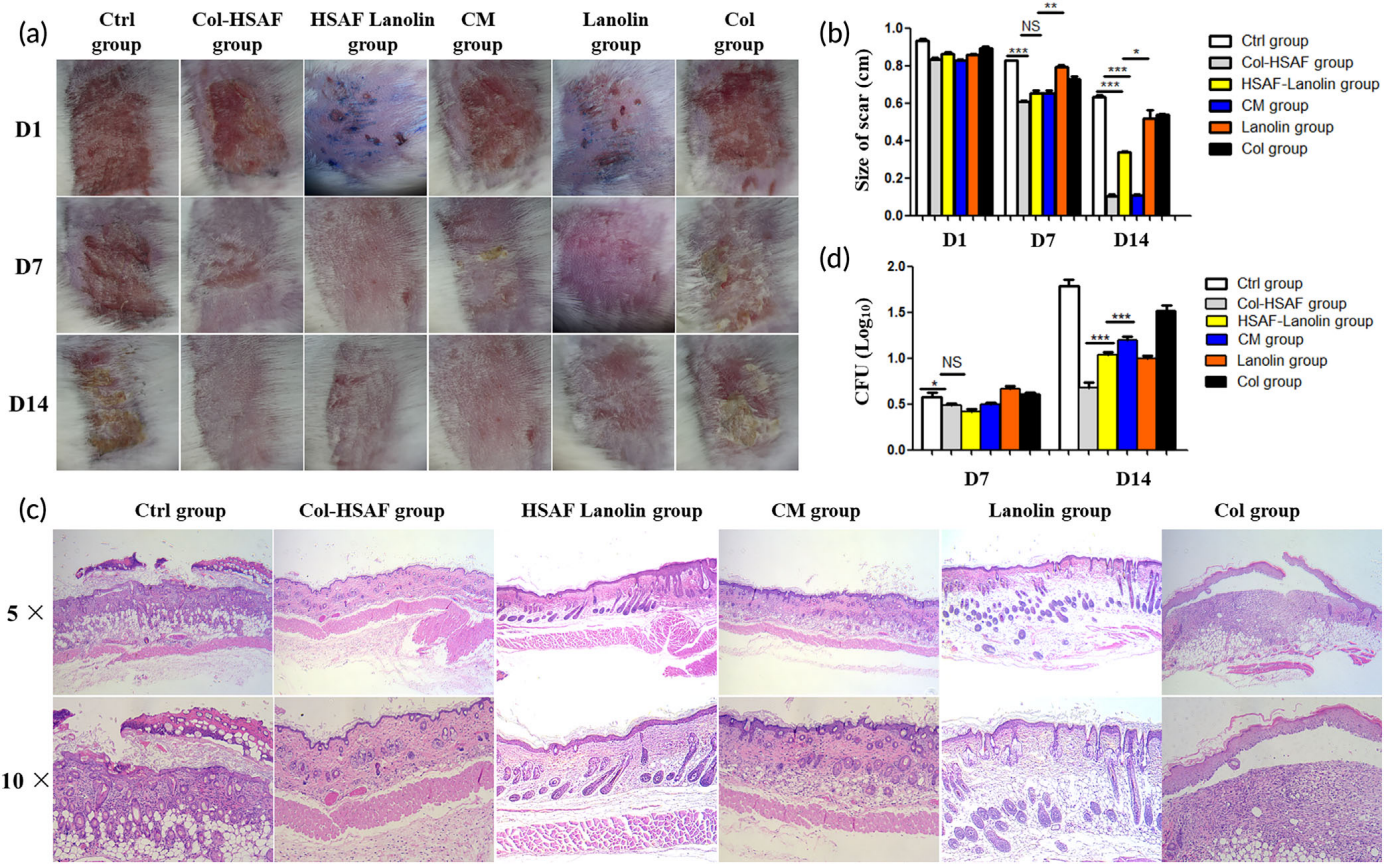
(a) Representative images of the skin and (b) size of the scar are shown to indicate disease progression in the Ctrl, Col‐HSAF, HSAF‐lanolin, CM, lanolin and Col groups in the FK animal model at 1, 7 and 14 days. (c) The histopathology of the Ctrl, Col‐HSAF, HSAF‐lanolin, CM, lanolin and Col group at 7 and 14 days. (d) Viable fungal loads reported in log colony‐forming units (CFU) in the Ctrl, Col‐HSAF, HSAF‐lanolin, CM, lanolin and Col group. Magnification: 10× (kin photographs) and 10× and 20× (HE staining). NS, not significant, *p < 0.05, **p < 0.01, and ***p < 0.001, *n* =5 animals per group. All data are presented as means ± SEM, and statistical significance was determined using paired *t* tests

In the cutaneous candidiasis mouse model, the antifungal activities of Col‐HSAF, HSAF‐lanolin, clotrimazole, lanolin, and Col were compared. As clearly indicated, all animals responded to treatment showing cessation of the propagation of fungal infection with gradual recovery along the study period. However, Col‐HSAF exhibited a comparable antifungal effect: the tested mice showed a prompt response to the treatment beginning on the first day of the treatment, in contrast to the other studied groups. Complete healing of the skin was observed in infected mice treated with Col‐HSAF, in which the size of scar was 0.63 ± 0.01 cm, 0.10 ± 0.01 cm (*p* < 0.001), 0.34 ± 0.01 cm (*p* < 0.001), 0.11 ± 0.01 cm (*p* < 0.05), 0.52 ± 0.05 cm and 0.54 ± 0.01 cm in the Col, Col‐HSAF, HSAF‐lanolin, clotrimazole, lanolin, and Col groups, respectively (Figure 7a,b).

Histological micrographs of the skin sections are displayed in Figure 7c. A microscopic examination of the untreated mice infected with *Candida* in the Ctrl group revealed a denuded dermis with ulcerated epidermis involving all the epidermal cell layers at the site of infection. The dermis showed severe edema, loose connective tissue, atrophic hair follicles, and mild inflammatory cellular infiltrate at the base of the ulcerated area. Infected skin treated with Col‐HSAF and with the market product clotrimazole exhibited ulceration at the site of infection with the formation of granulation tissue showing mild to moderate inflammatory cell infiltration. Severe edema of the dermis with atrophy of hair follicles and mild degeneration of the sebaceous glands were also observed. The skin of infected mice treated with Col did not show obvious improvement. This prominent efficacy was manifested by significant reductions in the count of fungal colonies remaining in the Col‐HSAF (0.49 ± 0.02), HSAF‐lanolin (0.42 ± 0.03), clotrimazole (0.50 ± 0.02) groups compared with Ctrl (0.58 ± 0.04), lanolin (0.66 ± 0.03), and Col (0.61 ± 0.02) groups at 7th day and was 1.79 ± 0.07, 0.68 ± 0.06 (*p* <0.001), 1.04 ± 0.30 (*p* <0.001), 1.20 ± 0.04 (*p* <0.001), 1.00 ± 0.03, and 1.52 ± 0.06 in the Ctrl, Col‐HSAF, HSAF‐lanolin, clotrimazole, lanolin, and Col groups (Figure 7d).

## 3 DISCUSSION

HSAF, a promising antifungal agent used in the biological control of fungal diseases of plants, was used as an antifungal agent in animal models of superficial fungal infection in this study. Three important points of innovation are reported: (1) HSAF, a potential antifungal agent for the treatment of superficial fungal infection, was extracted from marine‐derived *L. enzymogenes* YC36. The efficient and high‐purity extraction strategy achieved HSAF purity of 95.3%. (2) We developed an innovative antifungal agent for superficial therapy, and Col‐HSAF ophthalmic agents were constructed by using collagen as a carrier to overcome the antifungal's cytotoxicity and low solubility. (3) The effective therapeutic concentration of Col‐HSAF against superficial fungal infection was explored, and Col‐HSAF showed good biocompatibility and antifungal activities in animals models of *A. fumigatus* keratitis and cutaneous candidiasis.

Previously, HSAF was isolated from two terrestrial strains, *L. enzymogenes* C3 and OH11.^6^, ^7^ Although there have been many published studies on the biosynthetic mechanism of HSAF, there are relatively few studies on its regulation and bioengineering. Strains capable of producing HSAF is limited, so it is important to explore new strains capable of producing HSAF in special environments. In this study, we found that *L. enzymogenes* YC36 isolated from marine environments can inhibit the growth of many fungi and bacteria by producing abundant secondary metabolites. The whole genome of *L. enzymogenes* YC36 contains 15 biosynthetic gene clusters of secondary metabolites, including the antifungal compound HSAF.^18^, ^19^ In contrast to other terrestrial HSAF‐producing bacteria, in *L. enzymogenes* YC36, HSAF production is improved by regulating the NaCl concentration in nutrient‐deprived environments.

In addition, the majority of HSAF is lost during purification because of its poor solubility. A previous study showed that HSAF production is increased by 17.95% using a two‐stage temperature control strategy and adding glucose improve the yield of HSAF.^8^, ^20^ Moreover, the deletion of c‐di‐GMP genes from *L. enzymogenes* OH11 improves the yield of HSAF.^21^ In this study, we found a new method to increase the expression of the HSAF gene cluster and the yield of HSAF by adding the exogenous interspecific signaling molecule indole. Previously, indole has been widely studied in bacterial resistance and other bacterial social activities.^17^, ^22^ However, there are relatively few studies on the effects of secondary metabolite production. Here, we proved that an indole‐mediated increase in HSAF production is regulated by the two‐component system QseC/QseB.

HSAF is a highly potent antifungal compound, but it is cytotoxic and poorly soluble in water. In the wider drug release literature, release from hydrogel polymers is a popular and safe approach.^23^, ^24^ Therefore, we chose to use a Type I collagen membrane as the HSAF carrier, which combines good biocompatibility with high light transmittance and mechanical strength, ensuring that it will not significantly affect vision after being attached to the ocular surface. Collagen is a fibrous protein found in all multicellular animals and serves as a major constituent of many connective tissues. The administration of collagen to patients has also been reported to be safe and has been used as a biomaterial for antifungal drug delivery.^25^, ^26^ Sustained release of HSAF from Col‐HSAF was observed. On the one hand, HSAF was insoluble in water and contains hydrophobic groups in its molecular structure. It might interact with the hydrophobic groups on the collagen chain such as tyrosine, phenylalanine, and tryptophan during the process of collagen film formation, and this molecule was not easy to dissolve in the water again in an aqueous solution. On the other hand, after the collagen was dried and a film formed, the structure of Col‐HSAF was dense and the swelling rate in water was limited. The HSAF on the surface layer was released fast, while the HSAF on the inner layer was wrapped in the layered collagen and was difficult to diffuse. The antifungal activity of HSAF has been investigated in a previous study^5^, which suggested that HSAF disrupts the polarized growth of the fungus. Col‐HSAF showed good antifungal reagents in vitro in which the fungi were obviously inhibited and exhibited the highest sensitivity at MIC =1 μg/mm^2^, and *Aspergillus* spore growth was found to be controlled under transmission electron microscopy. Thus, the Col‐HSAF drug formulation and dosing regimen could be developed to maximize the therapeutic effect.

This in vivo study supports the therapeutic efficacy of the antifungal effects of Col‐HSAF in superficial infection of the skin and cornea in the mouse model. In the *A. fumigatus* keratitis model, Col‐HSAF obviously exerted better therapeutic effects than the Ctrl, Vor, and Col, as well as the HSAF β‐Cyclodextrin, and exerted a comparable effect to Col‐Vor. Col‐HSAF showed excellent biocompatibility and does not require frequent re‐application, even for severe infection, due to the controlled‐release characteristic of collagen. This effect differs from voriconazole, which is a triazole antifungal medication commonly used to treat *A. fumigatus* infections, and patients with aspergillosis must take this medication for several months or more, while inducing risks of serious side effects, such as transient visual disturbances, fever, rash, vomiting, diarrhea, peripheral edema, and respiratory disorder, which may increase due to the prolonged course of treatment.^27^ In the cutaneous candidiasis model, HSAF also exhibited excellent antifungal activity against *C. krusei* with obvious therapeutic effects on alleviating the skin swelling and inflammation at the infection site; these effects were comparable to those of clotrimazole, one of the azole antifungals used most commonly to treat cutaneous candidiasis. Topical forms of clotrimazole also carry the risk of serious side effects, such as contact allergic dermatitis, and the delivery may induce complications including nausea, vomiting, unpleasant mouth sensations, pruritus, and elevation of liver enzymes^28^, while topical Col‐HSAF could avoid these adverse reactions. Further studies of Col‐HSAF should focus on comparing therapeutic effects, adverse effects, solubility, and the cost benefit with those of additional existing commercial drugs in different fungal infection disease models with diverse fungal types. These studies would more comprehensively evaluate the role of HSAF as a clinical treatment for fungal infections and expand its application context. Col‐HSAF is a clearly a promising antifungal compound for treating superficial fungal infections.

## 4 MATERIALS AND METHODS

### 4.1 Strains and general methods

The strains used in this study are shown in Table S1. *L. enzymogenes* strains and the derived mutants were grown in LB medium and 40% TSB medium, and the optimal incubation temperature was 28°C. *F. solani* (CGMCC; Beijing, China), *A. niger* (CGMCC; Beijing, China), and *A. fumigatus* (CGMCC; Beijing, China) were grown in potato dextrose agar (PDA) medium (Hopebiol) and cultivated at room temperature. *C. krusei* was grown in Sabouraud dextrose agar (SDA) medium (Hopebiol) and cultivated at 26°C. *E. coli*, *P. aeruginosa* PAO1, and *B. subtilis* were grown in LB medium, and the optimal incubation temperature was 37°C.

### 4.2 Bioinformatics analysis

The genome sequence of *L. enzymogenes* YC36 was downloaded from NCBI (http;//www.ncbi.nim.nih.gov/) and uploaded to https://antismash.secondarymetabolites.org. Primers for real‐time PCR were designed by Premier 5.

### 4.3 Resistance test

*L. enzymogenes* YC36 has an inhibitory effect on *F. solani* ATCC 36031, *A. niger* ATCC 16404, *A. niger* CMCC 98003 (F), *A. fumigatus* AS 3.1320, *C. krusei* ATCC 14243, *E. coli* ATCC 25923, *P. aeruginosa* PAO1 and *B. subtilis* 168. *L. enzymogenes* YC36 was cultivated in 40% TSB for 1 day. Holes were punctured in an Oxford Cup containing PDA/SDA culture in a flat plate, and 100 μl of a bacterial solution containing *L. enzymogenes* YC36 was added to the holes. Then, the fungi or bacteria were directed toward the corresponding positions on both sides of the holes. Finally, the bacteriostatic cycle was measured.

### 4.4 Extraction of HSAF

Pure HSAF was purified by macroporous resin (Solarbio) adsorption and HPLC (Thermo Scientific Dionex Ultimate 3000 instrument; YMC‐Pack Pro C18 250 × 10.0 mm, S‐5 m, 12 nm). *L. enzymogenes* from −20°C was grown in LB medium at 28°C for 2 days. Bacteria on the plate were washed with saline water and transferred to LB medium for 1 day. Afterward, 2.5 ml of bacterial liquid were transferred to 50 ml of 40% TSB and cultivated for 3 days. After centrifugation, the macroporous resin was added to the supernatant for 1 day. Then, the macroporous resin was washed with 30%, 50%, 60%, and 100% methanol (Macklin). The 100% methanol eluent was vacuum concentrated, and the residue was dissolved with methanol and a small amount of methylene chloride. The crude extract was obtained by concentrating again. The residue was dissolved in methanol and dimethylsulfoxide (DMSO; Macklin) at a ratio of 1:2. Finally, an aliquot (95 μl) of each crude extract was purified using HPLC. The following chromatography conditions were used: solvent A: water containing 0.1% formic acid (Macklin); solvent B: acetonitrile (Merck‐Millipore) containing 0.1% formic acid; flow rate: 4 ml/min; detection wavelength: 320 nm; 44% solvent B increased to 100% at 12 min and 44% at 15 min. A small amount of pure HSAF was dissolved in 1 ml of methanol. High‐resolution mass spectrometry (HRMS) was carried out on an Agilent Q‐TOF 6520 mass spectrometer (Agilent).

### 4.5 Detection of HSAF

*L. enzymogenes* YC36 was grown in 40% TSB medium supplemented with different concentrations of NaCl and indole (Solarbio) for 2 days and grown at different times. Then, the liquid of bacteria was centrifuged. The supernatant was vacuum‐concentrated and dissolved in approximately 2 ml of methanol. Afterward, the liquid was concentrated again and dissolved in 0.5 ml of methanol and 1 ml of DMSO. Finally, an aliquot (5 μl) of each crude extract was analyzed using HPLC (Thermo Scientific Dionex Ultimate 3000 instrument; YMC‐Pack Pro C18 250 × 4.6 mm, S‐5 m, 12 nm). Indole was not added to the Ctrl group. For semiquantification, the peak areas of HSAF and its analogs were measured to obtain the relative yields of the compounds. The following chromatography conditions were used: solvent A: water containing 0.1% formic acid; solvent B: acetonitrile containing 0.1% formic acid; flow rate: 1 ml/min; detection wavelength: 320 nm; and solvent gradient from 20% B to 35% in the first 5 min, increased to 75% at 12 min, to 90% at 20 min, to 100% at 27 min, followed by 4 min with 100% B.

### 4.6 RNA extraction and quantitative real‐time PCR

*L. enzymogenes* YC36 was grown for 6, 12, 18, 24, 30, 36, 42, 48, 60 and 72 h. *L. enzymogenes* YC36 was grown in the presence of 0%, 0.5%, 1%, 1.5%, 2%, 2.5% and 3% NaCl. *L. enzymogenes* YC36 was grown in 40% TSB with or without indole. The Δ*qse*B and Δ*qse*C mutants were grown in 40% TSB with or without indole. Then, RNA was extracted from the above 20 bacterial liquid samples by a bacterial RNA kit (Omega). Reverse transcription was performed using the AccuRT Genomic DNA Removal Kit (ABM Good, Vancouver, Canada). The total reaction volume of quantitative real‐time PCR (TLIRBO) was 20 μl, including 2 μl of primers, 10 μl of SYBR green PCR master mix, 0.2 μl of ROX Reference Dye, 7.8 μl of cDNA template, and RNase‐free water. The reference gene was the 16S rRNA. The relative gene expression level was calculated as follows: ΔΔCt = ΔCt_(test)_−ΔCt_(calibrator)_, ΔCt_(test)_ = Ct_(target, test)_−Ct_(reference, test)_ and ΔCt_(calibrator)_ = Ct(target, calibrator)−Ct(reference, calibrator). A StepOne real‐time PCR System (AB Applied Biosystems) was used for real‐time PCR. The program was preincubated at 95°C for 5 min, and the program was run at 95°C for 20 s, at 55°C for 15 s, and at 72°C for 30 s for a total of 40 cycles. After 2 h, StepOne software V2.2 was used to analyze the cycle threshold (CT) and melting curve of each reaction. The primers used for qPCR are listed in Table S2.

### 4.7 Growth curve measurement

*L. enzymogenes* YC36 was grown for 16, 20, 24, 28, and 32 h in 40% TSB with or without indole. Then, the absorbance of bacteria was measured at 600 nm (OD_600_) and was using a UV spectrophotometer (Gene Quant 100).

### 4.8 Synthesis of Col, Col‐HSAF, Col‐Vor, Vor, sulfobutylether‐β‐cyclodextrin‐HSAF, and HSAF‐lanolin ointment

HSAF was dissolved in methanol at room temperature (2 mg/ml). Meanwhile, voriconazole (voriconazole for injection; Sichuan Medco Huakang Pharmaceutical Co., Ltd) was dissolved in ethyl alcohol and propylene glycol (1:1 in volume ratio) at room temperature (20 mg/ml). Then, HSAF solution and collagen I bovine protein solution were slowly mixed with different mass ratios of Col:HSAF = 25:2/25:1.5/25:1/25:0.5/25:0 at 4°C. The voriconazole solution and bovine collagen I protein solution were also slowly mixed at a mass ratio of Col:voriconazole = 25:2. The mixed solutions were stirred by using rotor‐magnetic stirring at 4°C for 24 h to make a homogeneous blend. The mixture was dispensed into a specific mold 5 mm deep and incubated in an incubator with a constant temperature and humidity (temperature, humidity: 30°C, 50%). After the samples were dried, membranes with different HSAF contents (2, 1.5, 1, 0.5, and 0 μg/mm^2^) and a constant voriconazole content (2 μg/mm^2^) were obtained.

Voriconazole solution (voriconazole for injection; Sichuan Medco Huakang Pharmaceutical Co. Ltd) was dissolved in ethyl alcohol and propylene glycol (1:1 in volume ratio) at room temperature (20 mg/ml). Then the dissolved Vor was diluted to 10 μg/ml with sterile normal saline.

Sulfobutylether‐β‐cyclodextrin was dissolved in water at a concentration of 2 mg/ml. Then, the HSAF solution was slowly added dropwise to the sulfobutylether‐β‐cyclodextrin solution at a mass ratio of HSAF to cyclodextrin of 3:10, and the mixture was stirred at room temperature for 2 h and allowed to stand for 30 min. Next, the solution was filtered with a 0.45 μm microporous membrane, and sulfobutylether‐β‐cyclodextrin‐HSAF was obtained after freeze‐drying.

For the preparation of the HSAF‐lanolin ointment, lanolin and the HSAF solution was mixed at a mass ratio of lanolin:HSAF = 1000:1.

### 4.9 Characterization of physical properties

Light transmission was measured with a UV3802 ultraviolet‐visible spectrophotometer (Shanghai UNICO) at 37°C over a range from 400 to 800 nm. Before testing, samples were immersed in deionized water for 1 h to absorb water completely, and the samples were then cut into rectangles. The tensile strength was determined with a uniaxial load testing instrument (Model #5567; Instron Corporation) equipped with a load cell of 10 N (Newton) capacity at a crosshead speed of 10 mm/min and an initial grip separation of 10 mm. The deionized water equilibrated samples were cut into dumbbell‐shaped specimens of identical rectangular gauge areas (width: 5 mm; gauge length: 10 mm) with two 8 mm end tabs. Thickness was measured with a thickness gauge. To avoid the breakage and slippage of samples in the jaws, there were 8 mm wide tabs on the end of each dumbbell. Samples were not stress preconditioned before failure testing was performed. During the tests, the samples were hydrated. Every reported value is an average of 5+ measurements. Then, Col‐HSAF and Col membranes were mounted on aluminum stubs directly and sputter coated with platinum for 70 s before examination by scanning electron microscopy (EVO18; Zeiss) to observe the structure.

### 4.10 In vitro drug release of HSAF

HSAF content was determined by an ultraviolet‐visible UV3802 spectrophotometer (Shanghai UNICO). The scans were registered from 200 to 400 nm. The detection wavelength of HSAF was determined after determining the characteristic absorption peak of HSAF. Then, the appropriate amount of HSAF was diluted with methyl alcohol to obtain final solution concentrations of 3.75, 7.5, 15, 30, 45, and 60 μg/ml. The absorbance was measured at the detection wavelength, and the mass concentration was used to draw a standard curve of the absorbance. The release rate of HSAF loaded in Col‐HSAF was determined by immersing Col and Col‐HSAF in 6 ml of a PBS solution and constantly shaken in a shaker at 37°C. The shaking speed was 60 rpm. At predetermined time intervals, 4 ml of drug release medium were sampled and detected, while 4 ml of fresh PBS pre‐equilibrated to 37°C were added to replace the removed drug release medium. The released HSAF was evaluated by measuring the UV–vis absorbance at 320 nm.

### 4.11 Cell viability and cell apoptosis assay

Human corneal epithelial cells (HCECs) were obtained from the American Type Culture Collection (ATCC) and cultured in Dulbecco's modified Eagle's medium supplemented with Nutrient Mixture F‐12 (Gibco BRL), 10% fetal bovine serum (Gibco), 100 U/ml penicillin and 100 μg/ml streptomycin (Gibco). Cells were grown to confluency in 25 cm^2^ polystyrene tissue culture flasks at 37°C in 5% CO_2_ and 95% air, and confluent cells were subcultured every 2–3 days by trypsinization with trypsin/EDTA solution.

The effects of Col‐HSAF on the morphology and proliferation of HCECs were observed by coculturing HCECs with the collagen membrane and Col‐HSAF at a concentration of 2 μg/mm^2^ for 24 h in 24‐well tissue plates (BD, Japan), and cells were randomly divided into the following four groups: the Ctrl group, 2 μg/mm^2^ Col‐HSAF group, Col group, and positive control group, which was treated with 0.2 M NaCl. After 24 h, the proliferation of HCECs was quantitatively determined by a CCK‐8 assay at an OD value of 450 nm with a microplate reader (BioTek Instruments).

Cell apoptosis was assessed by flow cytometry using an annexin V‐fluorescein isothiocyanate apoptosis detection kit (BD) according to the manufacturer's instructions. Briefly, cells were pooled, washed, and resuspended in 500 μL of binding buffer, followed by the addition of 500 μl of annexin V‐fluorescein isothiocyanate and 5 μl of PI. Then, the cells were incubated at room temperature away from light for 15 min and were subsequently analyzed by flow cytometry (Beckman Coulter EPICS XL/MCL). Viable cells did not exhibit annexin V or PI staining, early apoptotic cells showed annexin V but not PI staining, and late apoptotic cells exhibited both annexin V and PI staining.

### 4.12 In vitro antifungal effect

The strains *A. fumigatus* AS3.1320 and *C. krusei* ATCC 14243 used in study were purchased from the Guangdong Microbiological Culture Collection Center (GDMCC; Guangzhou, China). The *A. fumigatus* strain was cultured on Sabouraud dextrose agar (Difco) at 30°C for 4 days, and *C. krusei* was cultured on yeast malt agar at 26°C for 3 days. The spores were harvested in 1 ml of sterile PBS, counted with a hemacytometer, and then diluted to a concentration of approximately 2 × 10^5^ colony‐forming units (CFU)/mL for *A. fumigatus* spore preparation (AFSP) to generate the animal model.

### 4.13 Biological activity assay

The MIC is the lowest concentration of a chemical that inhibits visible growth of fungi. The experiment was performed in 96‐well plates with serial two‐fold dilutions of HSAF across the columns. Each dilution was performed in duplicate. Each well contained approximately 5 × 10^6^ CFU/ml of the final concentration of fungi. Controls with fungi and medium alone were included to ensure the viability of the fungi and the sterility of the medium. The fungal plate was then incubated at 37°C for 16 h before recording the results. The plate of fungi was then incubated at room temperature for 1–3 days before recording the results.

### 4.14 Inhibition zone test

The previously prepared AFSP was shaken in a test tube; then, 300 μl of the spore solution was added to the agar culture plate, and the solution was spread evenly with an inoculating loop. Col‐HSAF membranes with different HSAF contents (concentrations of 0, 0.5, 1, 1.5, 2 μg/mm^2^) were trephined into 5 mm diameters and placed in the center of the agar plate. The size of the inhibition zone was observed by the camera after the plates were incubated at 28°C for 72 h.

### 4.15 Microscopic morphology assays of *A. fumigatus*

*A. fumigatus* was divided into two groups: the Col‐HSAF group (*N* =5) and the Ctrl group (*N* =5). After 72 h, *A. fumigatus* was fixed with 2.5% glutaraldehyde and dehydrated with gradient concentrations of ethanol. Then, the samples were mounted on aluminum stubs and sputter coated with platinum for 70 s before the examination.

### 4.16 Pharmacokinetic study of Col‐HSAF

Animals. New Zealand albino rabbits (Animal Center of Zhongshan Ophthalmic Center at Sun Yat‐sen University Guangzhou, China) weighing between 2 and 3 kg were raised for at least 1 week under standardized temperature (25–28°C), humidity (50%–60%), and light (12 h light–dark) conditions before the experiment. Animals were allowed free access to standard food and tap water before and throughout the experiment. All care and handling of rabbits adhered to the ARVO Statement for the Use of Animals in Ophthalmic and Vision Research, and procedures were approved by the Institutional Authority for Laboratory Animal Care (Guangzhou, China; approval ID: 2019–083).

Tissue dissection and sample collection. All rabbits (*N* = 72) were divided into two groups: the Col‐HSAF group and the Ctrl group. Col‐HSAF at a concentration of 2 μg/mm^2^ Col‐membrane and a diameter of 1 cm^2^ was inserted into the conjunctival sac of the right eye. Six animals from each group were anesthetized and euthanized by exsanguination with 3% Dorminal solution at the corresponding time points (3, 6, 24, 72, 168, and 336 h, *n* =6 animals/time point/group) after administration, and then the eyes were enucleated at 3, 6, 24, 72, 168, and 336 h and placed on ice. Aqueous humor samples (150 μl each) were collected using the paracentesis technique at each time point. The anterior segment was dissected to obtain samples of the conjunctiva and cornea. The cornea and conjunctiva of each animal were cut into a spiral tube, an 80% DMSO solution was added, and ball meson was added to the tube. The FastPrep‐24 5G sample processing system was used to homogenize the samples for 30 s at a speed of 6 m/s. The samples were centrifuged at 307g for 10 min at a controlled temperature of 4°C, and the supernatant was removed as the homogenate of the corresponding tissue.

Determination of the HSAF content using LC–MS/MS. The LC–MS system, including the Surveyor autosampler and MS pump, was purchased from Waters. Chromatographic separation was performed on an ACQUITY UPLC BEH C18 column (2.1 mm × 50 mm 1.7 m; Waters) coupled with a guard column at 40°C. The mobile phases consisted of solvent, 0.1% formic acid in water (A), and 0.2% formic acid in acetonitrile (B). The following stepwise gradient elution program was used at a flow rate of 0.40 ml/min: 90% A for 0–0.5 min, 90%–5% A for 0.5–1.0 min, 5% B for 1.0–3.0 min, and 5%–90% A for 3–5.5 min. The mass spectrometer was operated with an electrospray ionization (ESI) source in negative mode and the experiment was performed in multiple reaction monitoring (MRM) mode. The MRM transitions and parameters for HSAF were as follows: capillary voltage: 3.00 kV; sample cone voltage: 60 V; source temperature: 150°C; desolvation temperature: 350°C; desolvation gas flow rate: 650 L/h; cone gas flow rate: 150 L/h. Transitions were as follows: *m*/*z* 511.36 → 97.80, cone: 79; collision energy: 28. The LC system and mass spectrometer were controlled using UNIFI software (Waters). Data were processed by plotting the peak area versus the relative analyte concentration with a weighting factor of 1/*x*.

### 4.17 Col‐HSAF therapy for *A. fumigatus* keratitis

#### 4.17.1 Establishment of an FK model

C57BL/6J mice (6–8 weeks old) were purchased from Beijing Vital River Laboratory Animal Technology Co., Ltd. They were housed in an environment with a cycle of 12 h of light and 12 h of darkness at 20°C. No disease was found in these animals by slit‐lamp examination and indirect fundoscopy. Briefly, the mice were anesthetized by administering an intraperitoneal injection of pelltobarbitalum natricum (0.3%, 0.1–0.2 ml/10 g). Proparacaine hydrochloride (0.5%) was used topically for corneal anesthesia. When no response to corneal touching was observed, a tunnel was made in the corneal stroma of a mouse with a 30 G needle; next, 5 μl of a solution containing the AFSP inoculum (5 × 10^6^ CFU/eye) was injected into the tunnel with a 33 G needle. The injection was successful when most of the cornea turned white. Twelve hours after the inoculation, all mice were randomly divided into six groups: the Col‐HSAF group, β‐CD‐HSAF group, Vor group, Col‐vor group, Col group and Ctrl group. In the Col‐HSAF, Vor and Col groups, the Col‐HSAF, Vor and Col membranes were cut into fragments using trephine with a diameter of 2.5 mm and attached to the left eye of mice. Membranes were replaced once daily, whereas the right eyes were untreated. In the β‐CD‐HSAF group, HSAF was mixed with cyclodextrin at a concentration of 2 μg/mm^2^, and then instilled into the conjunctival sac once daily. PBS was instilled into the conjunctival sac of the Ctrl group once daily.

Then, the eyelids of the mice were sutured with 6–0 Proline to ensure that the membrane could stay on the surface of the mouse eyeballs. Col, Col‐Vor, and Col‐HSAF were applied to mice only once during the experimental period. The mice in β‐CD‐HSAF group were subconjunctivally injected with the β‐CD‐HSAF solution (HSAF, 3 μg/ml, 5 μl per time) each day after infection. The mice in Vor group received 10 μg/ml voriconazole topically four times per day for seven consecutive days. At 1, 3, 5, and 7 days after the operation, the mice were photographed and clinically scored before they were humanely euthanized. Eyes were enucleated and processed for histological examination and quantitative microbial culture. All mice were treated in accordance with the Council for Purpose of Control and Supervision of Experiments on Animals, Ministry of Public Health, China. Corneal infections were induced in the right eyes.

#### 4.17.2 Clinical scoring

The inoculated eyes were scored with a slit lamp at 1, 3, 5, and 7 days. A grade of 0 to 4 was assigned to the area of opacity, density of opacity, and surface regularity. The area of opacity was graded as follows: 1, 1%–25%; 2, 26%–50%; 3, 51%–75%; and 4, 76%–100%. Density of opacity was graded as follows: 1, mild clouding, the iris vessels, and pupils are clearly visible; 2, medium clouding, the outline of the pupil is visible; 3, uneven clouding, the structure behind is not visible; and 4, uniform clouding, the structure behind is not visible. Surface regularity was graded as follows: 1, mild irregularity; 2, stroma is edematous and the surface is raised or sunken; 3, stroma was obviously edematous or depressed or postelastic layer bulging; and 4, the cornea is perforated. The final score of each eye is the sum of the scores of the three categories.

#### 4.17.3 Histopathological examinations

Infected eyes were enucleated from euthanized mice, fixed in 4% paraformaldehyde, and then embedded in paraffin 3, 5, and 7 days after infection. Continuous 5‐μm sections were stained with hematoxylin‐eosin (HE). The histological structure and the degree of inflammation and fungal invasion were evaluated by light microscopy.

#### 4.17.4 *Aspergillus* CFUs

Whole corneas from the murine model of *A. fumigatus*‐induced FK were placed in 1 ml of sterile PBS and homogenized. Serial 10‐fold dilutions were performed and plated onto Sabouraud dextrose agar plates (Merck). The plates were then incubated at 37°C for 72 h, and the CFU number was determined by direct counting.

#### 4.17.5 Ex vivo fluorescence staining and two‐photon microscopy (TPM)

The mice were sacrificed on the 3rd day postinfection, and the cornea was penetratively cut from the eyeball and the iris was removed; the cornea was cut and spread radially on a glass slide, 100 g/L KOH was added, and the cornea was stained with 100 mg/L CFW (Sigma‐Aldrich) working solution for 20 min. The slide was then left to stand for 1–2 min before being examined by fluorescence microscopy (Eclipse 80i) using blue light excitation (300–400 nm for the emission wavelength with excitation at approximately 355 nm). Then, the slides were observed under TPM. The purified water/gel was dropped between the sample and the objective lens, then the dichroic mirror was switched to WF mode, and the output light band of the wide‐field imaging light source was selected. The fungal hyphae were identified from TPM at an excitation wavelength of 790 nm. A filamentous structure was considered to be a fungal filament if (i) it could be recognized under fluorescence microscopy and confirmed using TPM, in which hyphae had many branches and were bamboo‐structured and surrounded by inflammatory cells; (ii) it exhibited branching and septation; and (iii) it was found within the central area of the slide; otherwise, a negative result was recorded.

### 4.18 Col‐HSAF therapy for *C. krusei* infection

#### 4.18.1 Establishment of a *C. krusei* model

The dorsal skin of each mouse was shaved, and an area of approximately 1 cm^2^ was marked on the back. The marked area was infected with 1 × 10^6^ CFU/ml of the suspension by gently rubbing it on the skin with a sterile cotton‐tipped swab until visible fluid was no longer observed.^29^ Infection was produced under an occlusive dressing, and the infected area was covered with a sterile bandage held in place with extra adhesive tape for 24 h before treatment began. The treatment of the infected mice began 24 h after the infection was induced and was continued for 14 days.

The mice were randomly divided into six groups containing 20 mice each. Group 1 served as a control and received no treatment; Groups 2 and 3 received Col‐HSAF (size: 1 cm^2^, content: 200 μg) and HSAF‐lanolin (size: 1 cm^2^, content: 200 μg) respectively; an equivalent dose of the market product clotrimazole cream (content: 30 μg; Guangdong Hengjian Co., Ltd) was applied once daily to Group 4; and Groups 5 and 6 was treated with lanolin or the Type I hydrogel without drug, respectively. The specific usage is described below. In the Col‐HSAF and Col groups, the Col‐HSAF and Col membranes were cut into fragments using a trephine with a diameter of 2.5 mm^2^ and attached to the skin of mice; membranes were replaced once daily. In the clotrimazole group, the drug was smeared on the skin surface once daily. In the Ctrl group, PBS was smeared on the skin surface once daily.

#### 4.18.2 *C. krusei* CFUs

Swabs were collected from each infected area and placed in sterile tubes containing 5 ml of yeast malt agar during 7 days of treatment. Serial dilutions were prepared and then 1 ml of each dilution was inoculated into Petri dishes containing 10 ml of PDA. The inoculated plates were incubated for 24 h at 37°C, and the colonies were counted.

#### 4.18.3 Histopathological examination

On the 7th day postinfection, after taking skin swabs, the mice were sacrificed, and the tested part of the shaved skin was excised and fixed in 10% buffered formalin. The fixed skin tissues were processed and embedded in paraffin. Sections were cut from the paraffin block by microtome and stained with hematoxylin and eosin. The structure was observed under a light microscope (Leica, DM‐6000, Wetzlar, Germany) with an integrating camera to identify histopathological changes.

### 4.19 Statistical analysis

The statistical analyses were performed using SPSS version 21.0 (IBM). Differences in the clinical scores and fungal viability results were analyzed via one‐way ANOVA with the least significant difference post hoc test. Significant differences were defined as a *p* value <0.05.

In the pharmacokinetics study, noncompartmental pharmacokinetic parameters were calculated using the WinNonLin 8.1 software package. *C*_max_ in the aqueous humor, conjunctiva and cornea, and the *T*_max_ were estimated by performing a visual inspection of semilogarithmic plots of the concentration‐time curves. The AUC (0–336 h) was calculated using the linear‐trapezoidal rule, with extrapolation to infinity (AUC [0–∞]) from the last detectable concentration using the terminal elimination rate constant (ke) calculated from a linear regression equation of the final log‐linear part of the drug concentration‐time curve. The apparent *t*_1/2_ was calculated as *t*_1/2_ =0.693/ke.

### 4.20 Data availability

All data associated with this study are present in the paper or the Supplementary Materials.

## 5 CONCLUSIONS

In this study, we developed an effective mode of treatment by applying HSAF to superficial fungal infections. The marine‐derived *L. enzymogenes* YC36 strain contains the HSAF biosynthetic gene cluster, which we activated by administering the interspecific signaling molecule indole. An efficient extraction strategy was developed to significantly improve the purity of the antifungal. Then, we constructed a Col‐HSAF composite membrane to test the therapeutic effects of HSAF on fungal infections. Col‐HSAF exerted good therapeutic effects on two models of superficial fungal infection, fungal keratitis, and skin infection. In addition, Col‐HSAF addresses the problem of water insolubility, provides continuous release of the antifungal, and reduces the toxicity of HSAF.

## AUTHORS' CONTRIBUTIONS

YW and JY developed the overall research ideas and goals. XYY, QD, YXZ, and ZRB were responsible for the extraction and purification of HSAF. ZX, JHY, LLP, and XZ constructed Col‐HSAF. JZ, ZX, YCX, and DLH evaluated the clinical efficacy of Col‐HSAF in the fungal keratitis and *C. krusei* animal models. JZ, ZX, and ZX collected and analyzed clinical data. JZ, ZX, ZX, SW, XYY, YW, and JY wrote the manuscript.

## CONFLICT OF INTEREST

The authors declare no competing interests.

### PEER REVIEW

The peer review history for this article is available at https://publons.com/publon/10.1002/btm2.10304.

## Supporting information


Table S1
Table S2

Figure S1

Figure S2

Figure S3

Figure S4

Figure S5

Figure S6

Figure S7

Figure S8

## Data Availability

The datasets used and analyzed during the current study are available from the corresponding author on reasonable request.
