# Supplementary material for: Developing a new treatment for superficial fungal infection using antifungal Collagen‐HSAF dressing
Source: Bioeng Transl Med. 2022 Mar 10;7(3):e10304. doi: 10.1002/btm2.10304 (PMC9472023; doi:10.1002/btm2.10304)
Supplement: Supplementary file 1 — Table S1 Table S2 [file BTM2-7-e10304-s009.docx]

**Table S1.** The strains used in this study.

| Strains | Relevant characteristics | Source |
| --- | --- | --- |
| **Bactria** |  |  |
| *L. enzymogenes* YC36 | Wild-type | Laboratory collection |
| ΔqseB | The deletion mutant of *qseB* in *L. enzymogenes* YC36 | Laboratory collection |
| ΔqseC | The deletion mutant of *qseC* in *L. enzymogenes* YC36 | Laboratory collection |
| *E. coli* ATCC 25923 |  | China General Microbiological Culture Collection Center |
| *P. aeruginosa* PAO1 |  | Laboratory collection |
| *B. subtilis* 168 |  | Laboratory collection |
| **Fungi** |  |  |
| *F. solani* ATCC 36031 |  | China General Microbiological Culture Collection Center |
| *A. niger* ATCC 1640 |  | China General Microbiological Culture Collection Center |
| *A. niger* CMCC 98003 (F) |  | China General Microbiological Culture Collection Center |
| *A. fumigatus* AS 3.1320 |  | [Guangdong Microbial Culture Collection Center](http://www.baidu.com/link?url=ihQk65ACZcsNtoNj_m62b9e4NNkIXt0y3fsIsKRpTEu-b8mzuBNR1XXf-dDW79s5mNbg3FeK4Xuu_HY_niDXP2rF9f8sHxAdZrVEd4QQ1wW) |
| *C. krusei* ATCC 14243 |  | [Guangdong Microbial Culture Collection Center](http://www.baidu.com/link?url=ihQk65ACZcsNtoNj_m62b9e4NNkIXt0y3fsIsKRpTEu-b8mzuBNR1XXf-dDW79s5mNbg3FeK4Xuu_HY_niDXP2rF9f8sHxAdZrVEd4QQ1wW) |

**Table S2.** Primers used in this study.

| **HSAF genes** | **Primer (5’-3’)** |
| --- | --- |
| HSAF-FA hydroxylase family-up | CCGGACCGGCGCGAGTG |
| HSAF-FA hydroxylase family-down | TGCCGACGTTGACCTTG |
| HSAF-PKS/NRPS-up | TCAACTCCTACGGCCTCA |
| HSAF-PKS/NRPS-down | CAGCGGCAGTTCGCCGA |
| HSAF-FAD-dependent oxidoreductase-1-up | AGTGGCTCAACGGCACCG |
| HSAF-FAD-dependent oxidoreductase-1-down | TCAGGAACGGATGCAGG |
| HSAF-FAD-dependent oxidoreductase-2-up | AGAGCGTCGGCGAGAAG |
| HSAF-FAD-dependent oxidoreductase-3-up | ACCGGCTGGAAGCGCAC |
| HSAF-FAD-dependent oxidoreductase-3-down | TGAACGCCTCGCGCAGCAG |
| HSAF-alcohol dehydrogenase zinc-binding protein-up | GCACCTTCCCGATGGTGT |
| HSAF-alcohol dehydrogenase zinc-binding protein-down | TCCGGCCGAGCCGGCGGT |
| YC36_16S-up | TAGAGTGCGGTAGAGGGTAGCGGAA |
| YC36_16S-down | GTTCGCATCGTTTAGGGCGTGGACT |
