# Supplementary figures and images for: Developing a new treatment for superficial fungal infection using antifungal Collagen‐HSAF dressing
Source: Bioeng Transl Med. 2022 Mar 10;7(3):e10304. doi: 10.1002/btm2.10304 (PMC9472023; doi:10.1002/btm2.10304)

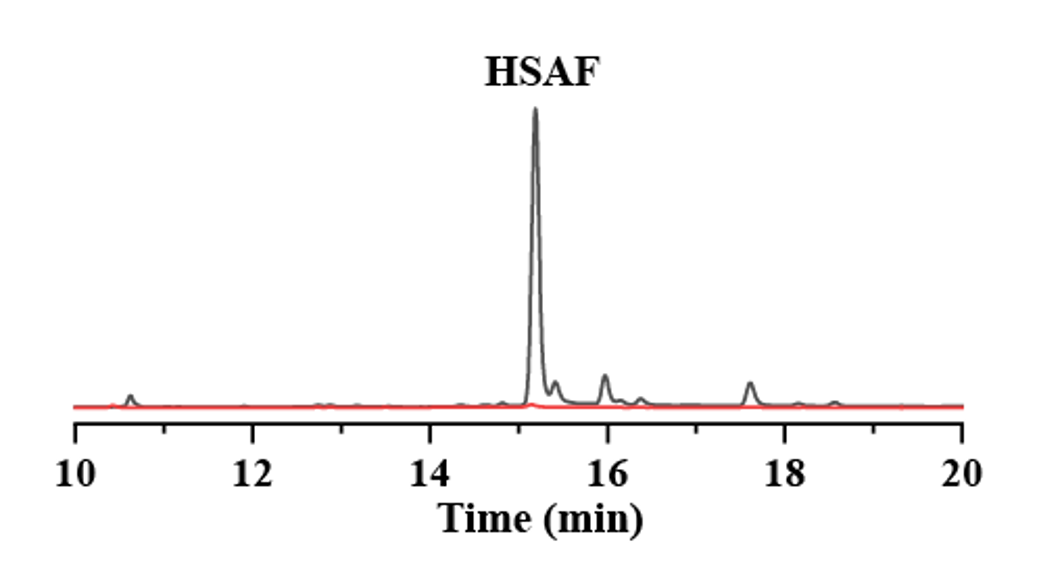

Supplement: Supplementary file 2 — Figure S1 [file BTM2-7-e10304-s007.png]

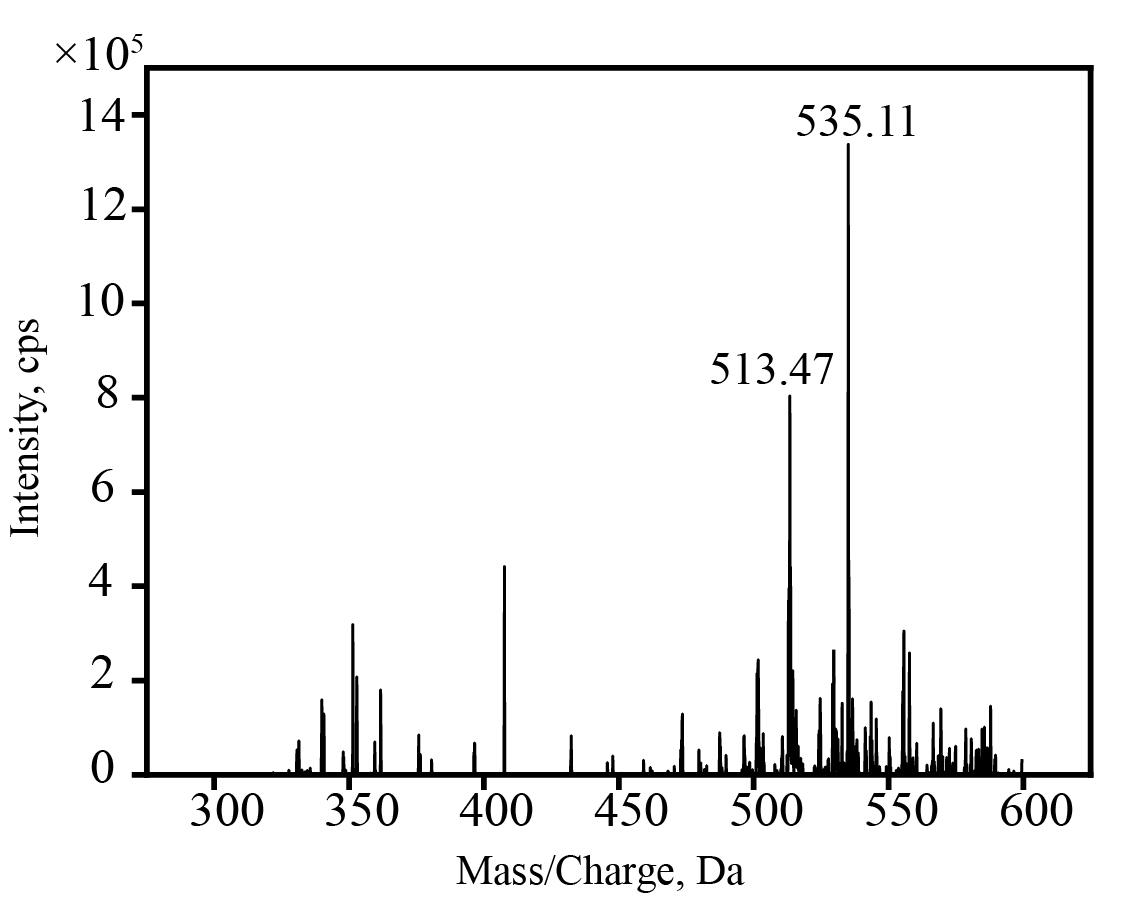

Supplement: Supplementary file 3 — Figure S2 [file BTM2-7-e10304-s006.jpg]

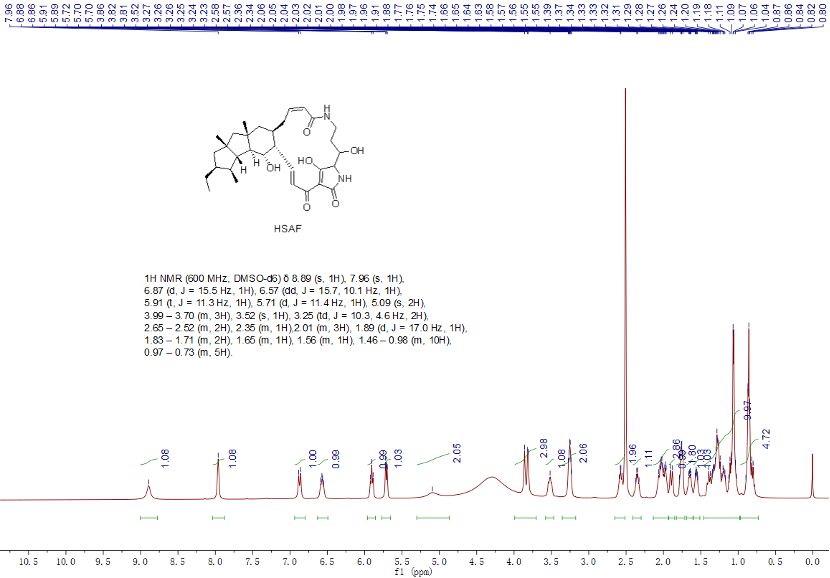

Supplement: Supplementary file 4 — Figure S3 [file BTM2-7-e10304-s005.png]

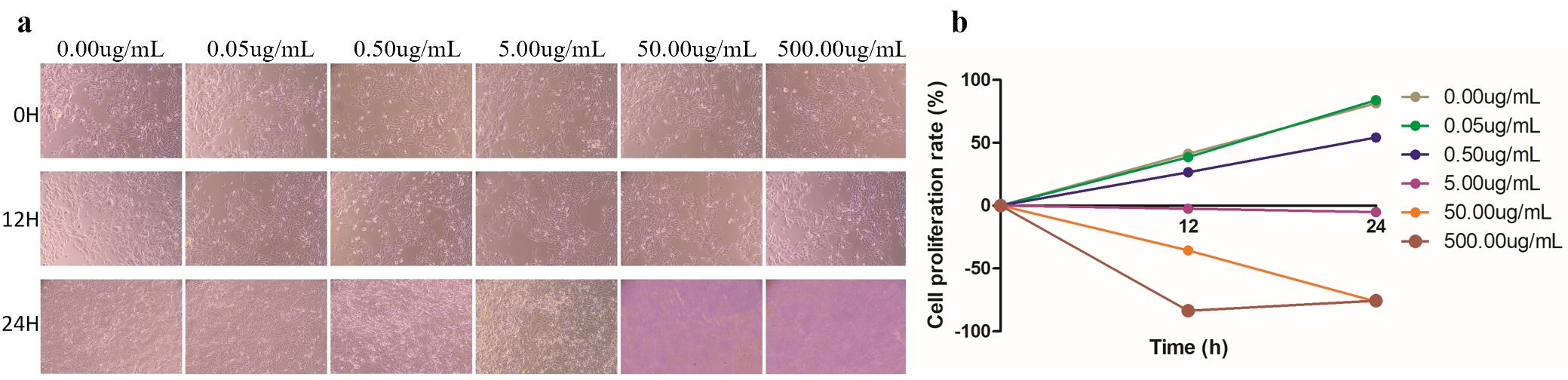

Supplement: Supplementary file 5 — Figure S4 [file BTM2-7-e10304-s002.png]

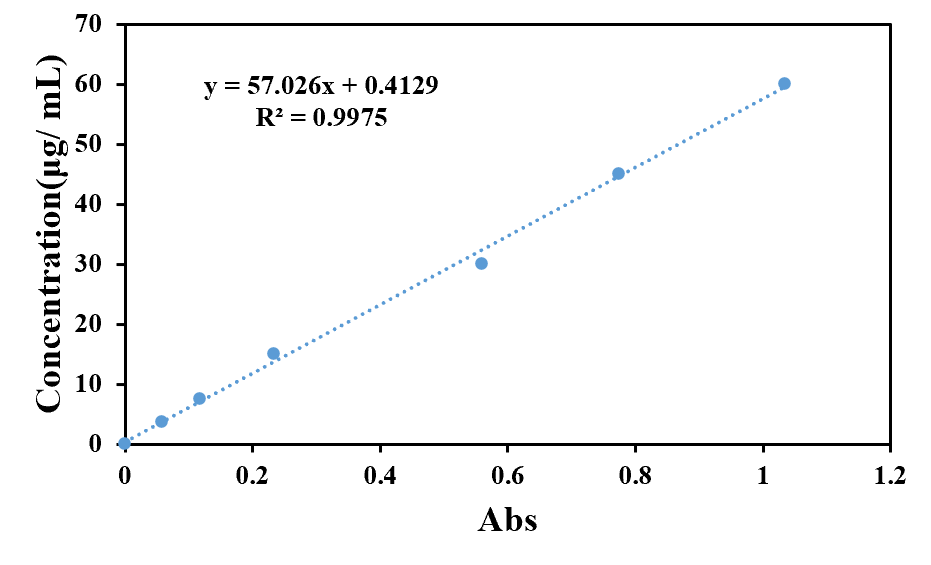

Supplement: Supplementary file 6 — Figure S5 [file BTM2-7-e10304-s001.png]

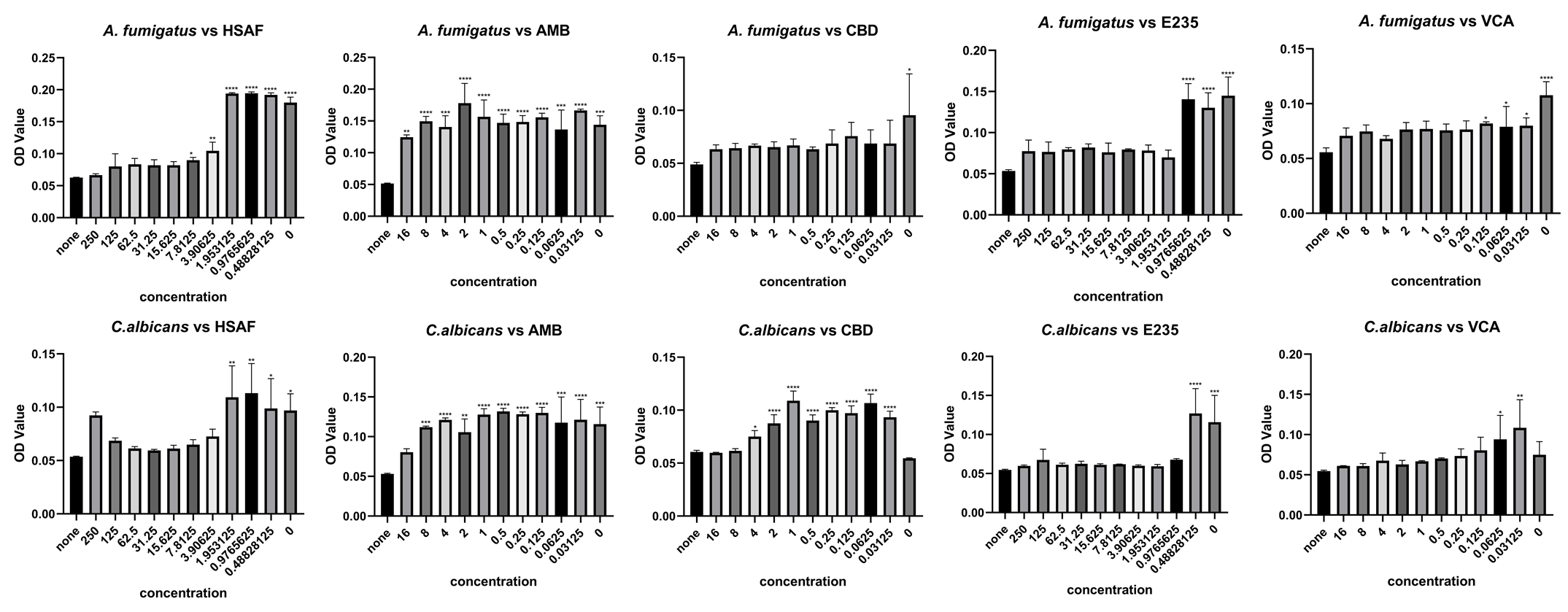

Supplement: Supplementary file 7 — Figure S6 [file BTM2-7-e10304-s008.png]

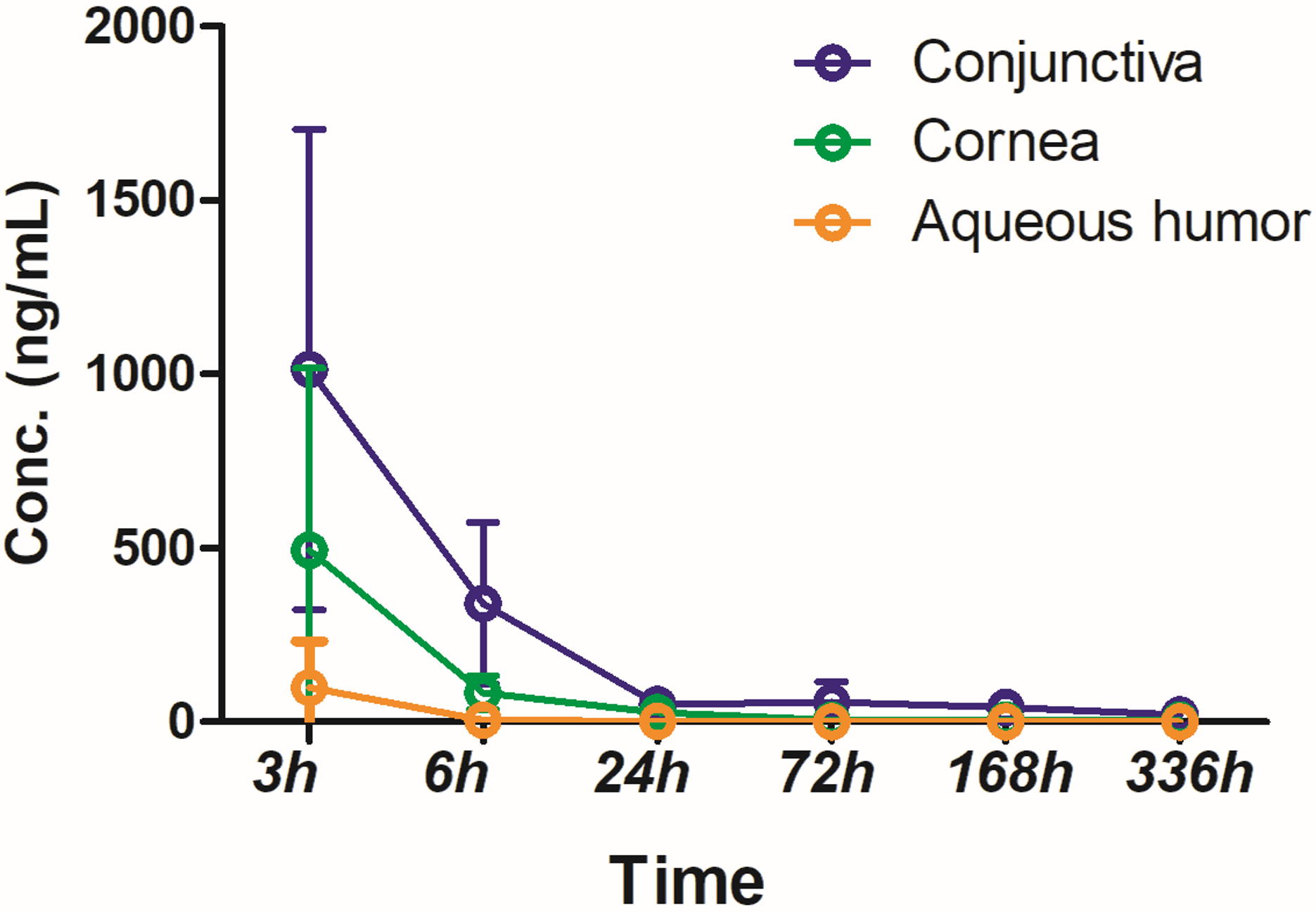

Supplement: Supplementary file 8 — Figure S7 [file BTM2-7-e10304-s003.png]

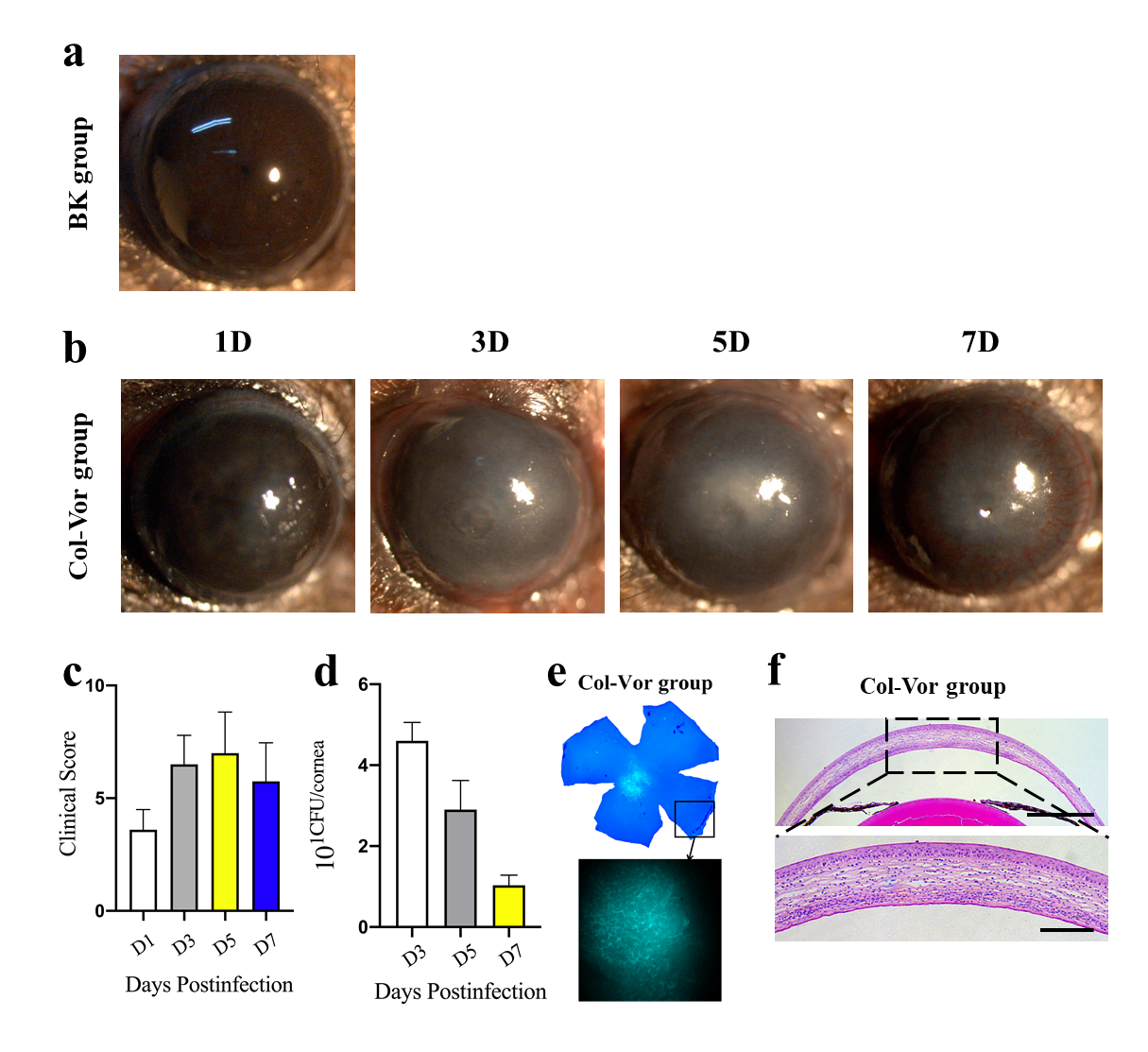

Supplement: Supplementary file 9 — Figure S8 [file BTM2-7-e10304-s004.png]
